# Morphology and swimming behavior of prezoeae in two stenopodidean shrimps: Functional implications for early larval life

**DOI:** 10.1371/journal.pone.0361633

**Published:** 2026-11-10

**Authors:** Mikihiro Nakagawa, Tomomi Saito

**Affiliations:** Usa Marine Biological Institute, Kochi University, Kochi, Japan; Sao Paulo State University Julio de Mesquita Filho: Universidade Estadual Paulista Julio de Mesquita Filho, BRAZIL

## Abstract

Most decapod crustaceans undergo a brief prezoeal stage immediately after hatching, during which the body remains enclosed within a thin embryonic cuticle. Although this stage typically lasts only minutes and is often omitted from larval descriptions, detailed morphological records exist for various taxa. However, these descriptions remain fragmentary, and studies linking morphology to behavior are almost nonexistent, leaving the biological and evolutionary significance of this short developmental stage largely unexplored. This study documents the prezoeal morphology and swimming behavior of two stenopodidean shrimps: *Spongicola venustus* (De Haan, 1844), a deep-sea sponge-associate, and *Stenopus hispidus* (Olivier, 1811), a shallow-water free-living species. The aim was to clarify phylogenetically conserved traits and the biological significance of morphology and behavior in relation to ecological type. Ovigerous females were collected from Tosa Bay, Japan, and reared in aquaria until hatching. Morphological observations were conducted on preserved specimens, whereas swimming behavior was analyzed using slow-motion microscopy movies. Both species hatched in the prezoeal stage, enclosed in an embryonic cuticle as observed in other taxa, with key zoeal features visible beneath. The prezoeae swam via rhythmic strokes of the antennae (scaphocerites), occasionally aided by pleonal flexions. Plumose setae on the antennae and telson appeared to enhance propulsion. These traits may reduce the risk of snagging by allowing elongated appendages to remain folded and may facilitate emergence from egg chambers or sponge mesh. They are also likely to be crucial for promoting initial ascent immediately after hatching. This swimming activity driven by cephalic appendages in prezoeae resembles naupliar locomotion, suggesting partial retention of primitive locomotor patterns. In addition, similar to insect pronymphs, the prezoeal stage may be effective in overcoming physical barriers associated with hatching, representing a response to shared selective pressures during early development. These findings provide new insights into the evolutionary diversity of developmental strategies in decapod crustaceans.

## Introduction

The prezoeal stage observed in decapod crustaceans (Pleocyemata) is defined as either the final stage of embryonic development or the first larval form immediately after hatching [1]. Morphologically, the entire body is enclosed within a thin membrane known as the embryonic cuticle. This stage lasts for an extremely short time, ranging from a few minutes to several hours, and cases have been reported in which prezoeae fail to molt and die following abnormal hatching under certain conditions, such as reduced salinity [2]. Consequently, whether the prezoea should be regarded as a true larval stage has long been a subject of debate [2,3]. The accumulation of detailed morphological descriptions, particularly for brachyuran crabs, in recent years has led to increasing recognition of the prezoeal stage as a regular phase of the life cycle [4,5].

Despite its brief duration, the prezoeal stage may represent a critical transition between embryonic and larval life, potentially influencing dispersal, locomotion, and survival immediately after hatching. Nevertheless, some review articles continue to describe it merely as the terminal embryonic stage [6], which indicates that its biological interpretation remains incomplete. The most critical issue in understanding the nature of the prezoea lies in elucidating its biological significance; however, studies explicitly addressing this question are extremely limited. Addressing this knowledge gap requires comparative analyses of prezoeal morphology and behavior across a wide range of decapod taxa. At present, available knowledge is strongly biased toward brachyurans, with information on carideans and other decapod groups remaining scarce.

In stenopodidean shrimps, the prezoeal stage was first described in members of the family Spongicolidae examined during the Challenger expedition (1872–1876) [7,8]. At that time, this stage was referred to by the unique term “brephalos”; however, subsequent reviews suggested that it corresponded to the prezoeal stage as currently defined [9–11]. Thereafter, fragmentary morphological observations of the prezoea have been reported for *Stenopus hispidus* [12] and *Microprosthema semilaeve* [13], including features such as a folded rostrum, well-developed second antennae, and poorly developed setation on the appendages. In addition, the direct-developing species *Spongiocaris japonica* has been shown to hatch as a prezoea-like form enclosed within an embryonic cuticle [14]. Taken together, these observations suggest that the prezoeal stage may be widely present across Stenopodidea [10,11]. Nevertheless, prezoeal morphology, swimming behavior, and the transition to the first zoeal stage have not been examined comprehensively in stenopodideans.

Members of Stenopodidea occupy habitats ranging from shallow coastal waters to the deep sea and exhibit lifestyles ranging from free-living to obligate symbiosis. Their contrasting habitats and life histories provide an opportunity to assess whether prezoeal morphology and behavior are evolutionarily conserved or shaped by ecological specialization. In particular, the deep-sea sponge-associated *Spongicola venustus* and the shallow-water free-living *Stenopus hispidus* represent contrasting ecological conditions under which the earliest post-hatching stage occurs. Understanding early life-history stages is therefore essential for elucidating their adaptive evolution and patterns of pelagic dispersal. We hypothesized that, despite their contrasting ecological niches, both species would exhibit conserved prezoeal morphological and behavioral traits that facilitate emergence, early locomotion, and initial dispersal, reflecting a common adaptive strategy during the transition from embryonic to larval life.

In this study, we compared the morphology and swimming behavior of the prezoeae of *Spongicola venustus* and *Stenopus hispidus*. In addition, we examined the effect of temperature on the timing of metamorphosis from the prezoea to the first zoea. Through detailed observations of stenopodidean prezoeae, this study provides a basis for understanding the biological significance of the prezoeal stage from the perspectives of evolutionary developmental biology and comparative behavioral biology, moving beyond the previously held view of a collection of isolated phenomena. This approach provides insight into survival strategies operating immediately after hatching in decapod crustaceans.

## Materials and methods

### Collection of ovigerous shrimps

Ovigerous females of *Spongicola venustus* were collected from glass sponges caught during two research cruises conducted in Tosa Bay, Japan. Sampling was carried out by the training ship “Toyohata-maru” in October 2024 using a trawl net (mouth width: 1.5 m; mesh size: 2.0 mm), and by the bottom trawling vessel “Tsukasa-maru” in February 2024 using a bottom trawl net. Ovigerous females of *Stenopus hispidus* were collected from a fishing port quay and rocky reefs using dip nets or by scuba diving between October 2022 and July 2025 in Tosa City and Otsuki Town, Hata District, Kochi Prefecture, Japan. The specimens of both species were maintained in rearing facilities at the Usa Marine Biological Institute, Kochi University. These shrimp species are not listed under any conservation laws, and collection was conducted under permission from Kochi Prefecture and relevant local fishery cooperatives.

### Adult rearing conditions

Adults of *Spongicola venustus* were maintained in a small tank (15.5 × 14.0 × 11.5 cm) placed within a 120-cm overflow aquarium under dark conditions (achieved via blackout curtains). Water temperature was maintained at 15–19°C, salinity at 28–34 ppt, and pH at 8.1–8.2. Adults of *Stenopus hispidus* were maintained in a standard aquarium tank (size of approximately 120 × 45 × 45 cm) at a water temperature of 24–26°C, salinity of 28–34 ppt, and pH 8.1–8.3. Lighting followed the ambient L:D cycle in the laboratory until prezoeae were released. Both species were fed a commercial crustacean feed paste (Itosui Ebi no Shushoku Nattokin).

### Morphological and behavioral observations

To evaluate temperature-dependent metamorphic rates, we measured the duration from hatching to complete transformation into the first zoea under two water temperatures for each species: 15°C and 20°C for *Spongicola venustus*, and 20°C and 25°C for *Stenopus hispidus*. Newly hatched larvae (*Spongicola venustus*: November 2024; *Stenopus hispidus*: June and July 2025) were fixed in sugar-formalin (20 g sucrose in 100 ml of 3% neutral buffered formalin). Body size was measured to the nearest 0.01 mm using an ocular micrometer under a stereomicroscope. Total length (TL) was defined as the linear distance from the anterior tip of the carapace along the midline to the posterior margin of the telson. Carapace length (CL) was defined as the linear distance between the anterior margin of the carapace at the base of the rostrum (excluding the folded rostrum) and the posterior margin of the carapace along the midline. Appendages were dissected under a stereomicroscope and sketched using a drawing device (Nikon Y-IDT) attached to a biological microscope (Nikon ECLIPSE E400). Shrimp morphology was sketched using a drawing tube (Nikon P-IDT) attached to a stereomicroscope (Nikon SMZ800). Lateral views and the metamorphic transition from the prezoeal to first zoeal stage were recorded using a digital camera (Nikon D5600) mounted on a stereomicroscope. Swimming behavior was recorded at 8 × slow motion using a smartphone camera (iPhone 11 and 15) mounted on the stereomicroscope. Image sequences were extracted and processed using Adobe Premiere Pro and subsequently subjected to color correction and trimming in Adobe Photoshop. The specimens are deposited in the National Museum of Nature and Science, Tokyo, Japan (NSMT-Cr 33690–33693).

## Results

### 1. Hatching prezoeae and transformation to first zoeae

At the time of collection, the shrimp eggs contained the blue yolk characteristic of the early stages of embryogenesis. During the late embryonic stage, this blue yolk was progressively absorbed, and a yellow yolk became visible through the developing embryos, with the eyes clearly discernible immediately prior to hatching. Hatching typically started a few hours after sunset and continued over two consecutive nights. In both species, all embryos hatched as prezoeae from the egg cases attached to the female’s pleopods (Fig 1A and 1B). Following hatching, the prezoeae immediately initiated upward swimming toward the surface (details of swimming behavior are described below) and ceased to do so after a short interval. Subsequently, the rostrum—initially folded beneath the carapace—began to extend, and the embryonic cuticle covering the cephalothorax, including appendages from the first antennule to the first pereopod, was gradually pulled away via the connecting membrane by extension movements of the pleon. Once the rostrum was fully extended, the cephalothoracic embryonic cuticle, which had been drawn posteriorly by the telson, detached. Finally, vigorous pleonal flexions caused the embryonic cuticle surrounding the telson to separate, allowing the previously folded processes and setae beneath the cuticle to expand completely, thereby completing the transformation into the first zoea. The process of transformation from prezoea to first zoea is presented in S1 Movie for *Spongicola venustus* and in S2 Movie for *Stenopus hispidus.*

**Fig 1:**
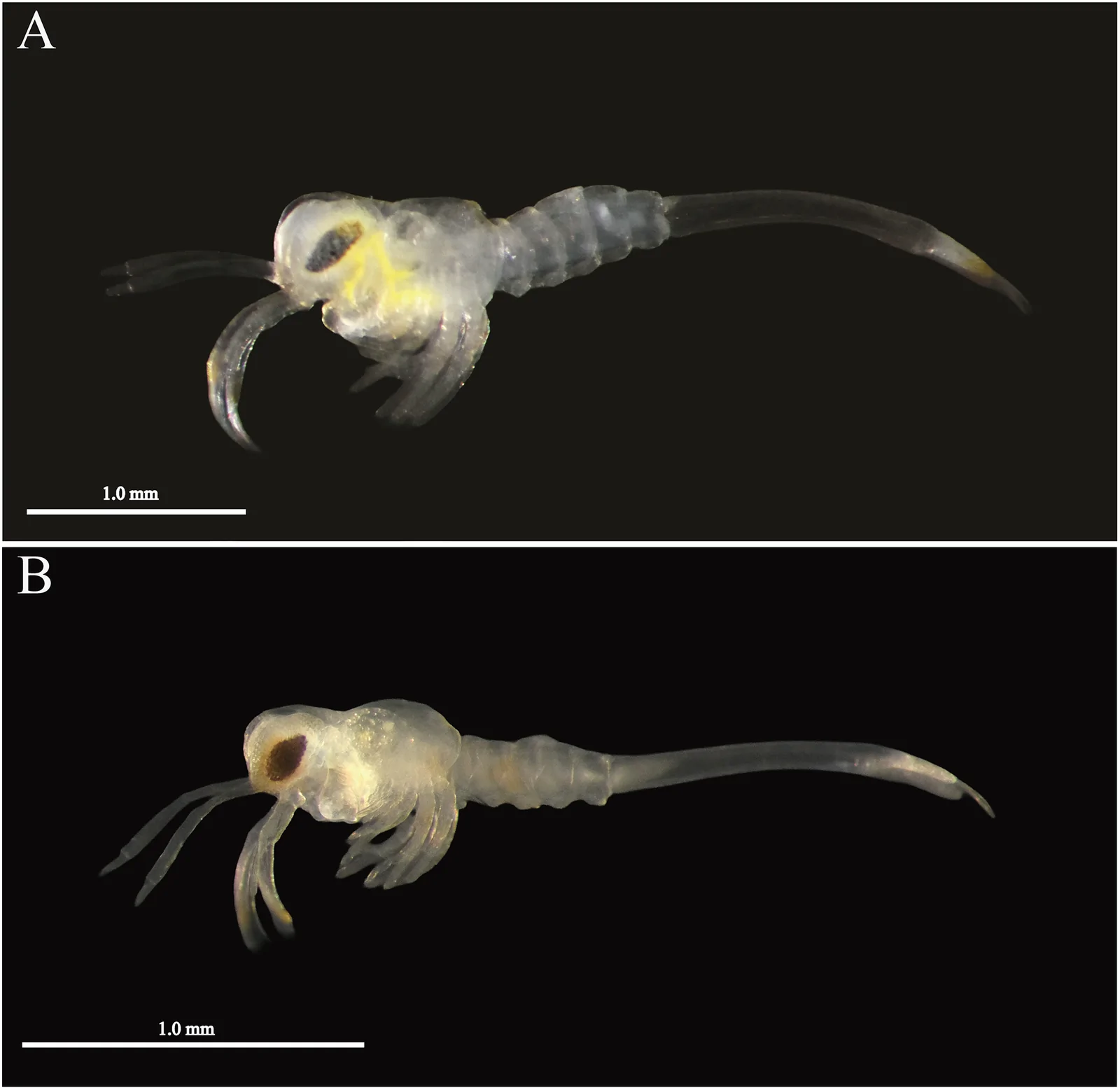
Hatched prezoeae of *Spongicola venustus* (De Haan, 1844) (A) and *Stenopus hispidus* (Olivier, 1811) (B).

The time required from hatching to completion of the molt into the first zoea varied significantly with temperature. In *Spongicola venustus*, the mean duration was 1802 ± 228 s at 15°C (n = 10) and 949 ± 209 s at 20°C (n = 20) (*p <* 0.05; Fig 2A), whereas in *Stenopus hispidus*, it was 887 ± 425 s at 20°C (n = 21) and 421 ± 150 s at 25°C (n = 24) (*p <* 0.05; Fig 2B).

**Fig 2:**
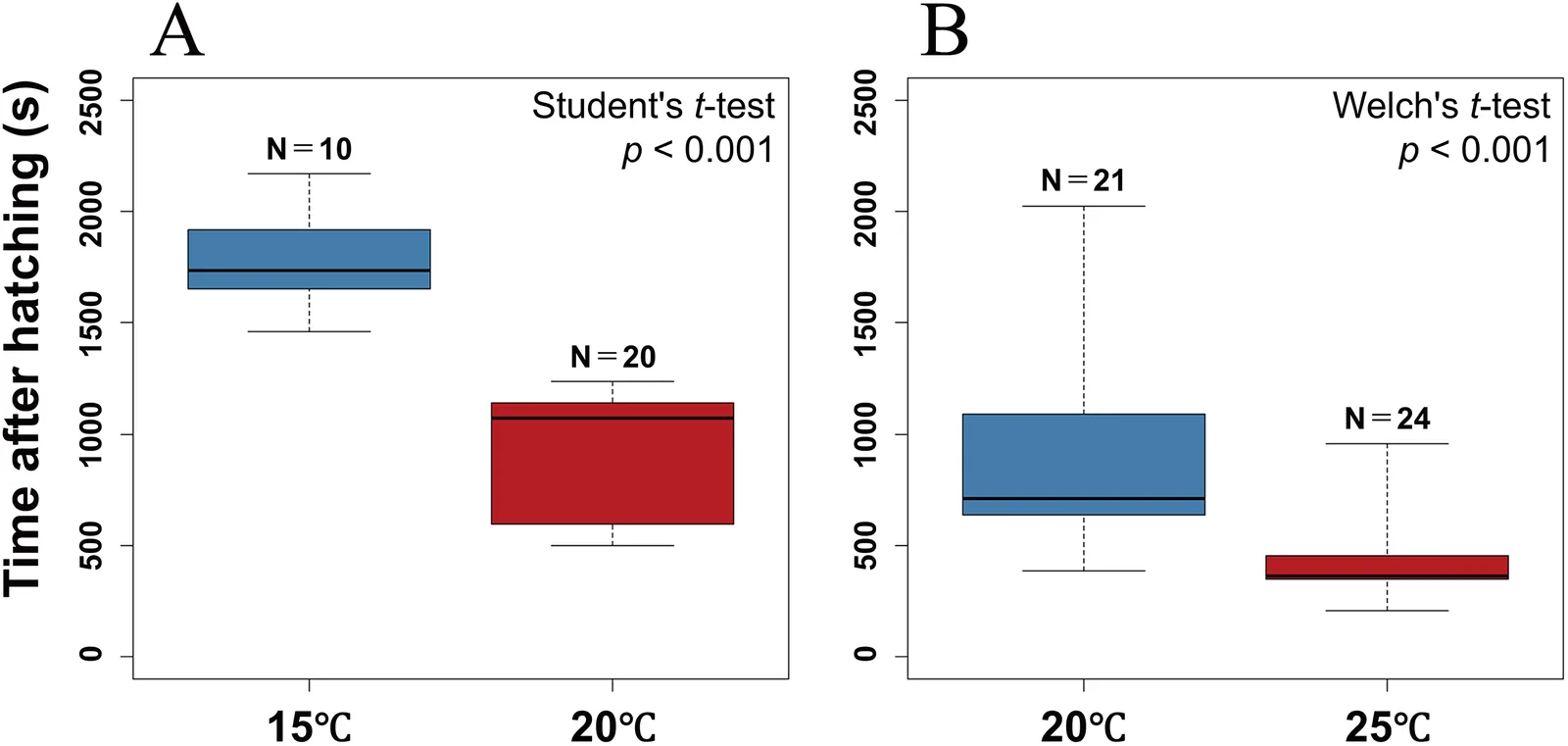
Duration of the prezoeal stage in *Spongicola venustus* (De Haan, 1844) (A) and *Stenopus hispidus* (Olivier, 1811) (B).

### 2. Morphological observations

The entire shrimp body is enclosed within a thin embryonic cuticle. The carapace is smooth and lacks projections, with the rostrum folded beneath it. Pleonal processes are folded ventrally, and the tergum is smooth. The mandible is laterally constricted. The setae of the thoracic appendages are partly embedded in basal musculature and remain undeveloped within the cuticle, whereas those on the antennae and telson are enclosed in hollow cuticular sheaths. Some setae present in the first zoea are not exposed at this stage, likely due to immaturity or concealment; therefore, the following description is based only on observable structures.

#### Description of the prezoeal stage in *Spongicola venustus* (De Haan, 1844) (Fig 3).

Dimensions: CL = 1.01 ± 0.03 mm; TL = 3.58 ± 0.07 mm (n = 5). Body entirely covered by a thin embryonic cuticle. Carapace smooth. Eyes sessile and pigmented. Rostrum folded beneath the carapace. Pleon possessed protrusions on the posterior ventral margins of the first to fifth somites, folded ventrally (Fig 3A and 3B). Antennule biramous with unsegmented peduncle; endopod bore one long seta, whereas exopod carried two setae and three aesthetascs (Fig 3C). Antenna biramous with unsegmented peduncle; endopod possessed two long plumose setae; exopod (scaphocerite) 7-segmented, bearing nine long setae, each enclosed within a hollow plumose embryonic cuticle (Fig 3D). Mandibles asymmetrical, with well‑developed incisor and molar processes (Fig 3E and 3F). Maxillule with a coxal endite bearing six setae and a basial endite bearing five setae, all enclosed within a hollow embryonic cuticle; endopod rudimentary (Fig 3G). Maxilla possessed a bilobed coxal endite with eight and three setae, and a bilobed basial endite with three and five setae; endopod with two setae; scaphognathite bore six marginal setae (Fig 3H). First maxilliped covered by an embryonic cuticle; coxa bore two setae, basis with eight setae; endopod 3‑segmented with 2, 2, and 4 setae proximally; exopod possessed four terminal setae, partially exposed (Fig 3I). Second and third maxillipeds also covered by embryonic cuticle; each endopod 5‑segmented; coxa and basis without seta; each exopod carried six setae (Fig 3J and 3K). First pereopod covered by an embryonic cuticle; coxa and basis with 0, 1 setae, respectively; endopod bore five setae; exopod carried six setae (Fig 3L). Telson with posterior margin bore seven pairs of processes an outermost spiniform pair, followed by five pairs of long setae, and an innermost short pair, first to sixth pairs enclosed within a hollow plumose embryonic cuticle (Fig 3M).

**Fig 3:**
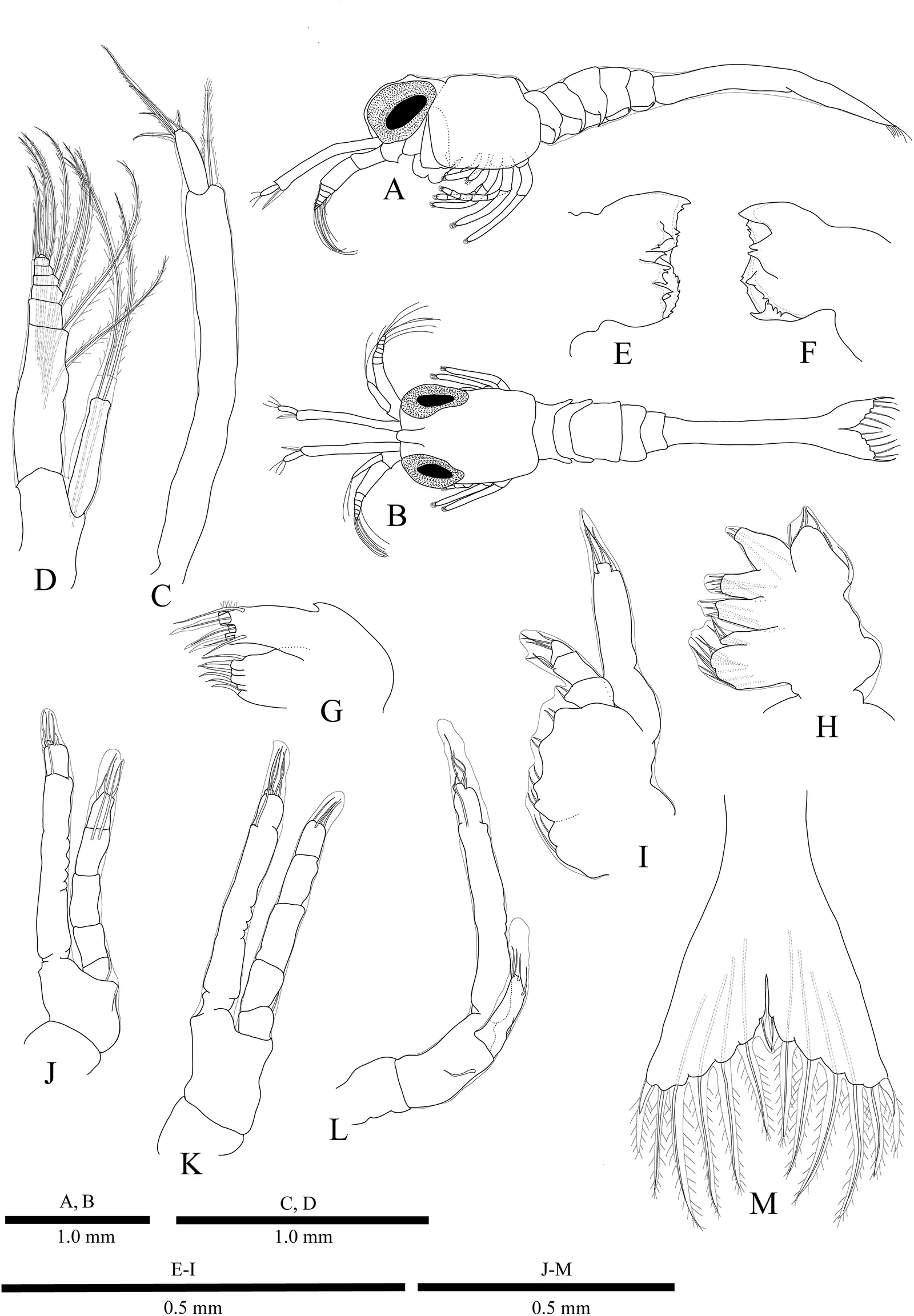
Prezoea of *Spongicola venustus* (De Haan, 1844). A: whole animal, lateral view; B: same, dorsal view; C: antennule; D: antenna; E, right mandible, posteroventral view; F, left mandible, posteroventral view; G: maxillule; H: maxilla; I–K: 1st–3rd maxillipeds; L: 1st pereopod; M: telson.

#### Description of prezoea in Stenopus hispidus (Olivier, 1811) (Figs 4 and 5).

Dimensions: CL = 0.82 ± 0.02 mm; TL = 2.92 ± 0.08 mm (n = 5). Body entirely covered by a thin embryonic cuticle, generally similar to that of *Spongicola venustus*. Carapace smooth. Eyes sessile and pigmented. Pleon possessed protrusions on the posterior ventral margins of the first and third somites, folded ventrally, and a dorsal spine on the third somite that remained folded beneath the embryonic cuticle (Fig 4A and 4B). Antennule biramous with 2‑segmented peduncle; endopod bore one long seta; exopod carried one seta and three aesthetascs (Fig 4C). Antenna biramous with an unsegmented peduncle; endopod possessed two long plumose setae and one short seta, whereas exopod (scaphocerite) 7-segmented, bearing nine long setae, each encased within a hollow plumose embryonic cuticle (Fig 4D). Mandibles asymmetrical, with well‑developed incisor and molar processes (Fig 4E and 4F). Maxillule with a coxal endite bearing six setae and a basial endite bearing five setae, all enclosed within a hollow embryonic cuticle; endopod rudimentary (Fig 4G). Maxilla possessed a bilobed coxal endite with four and three setae, and a bilobed basial endite with three and five setae; endopod carried four setae; scaphognathite bore five marginal setae (Fig 4H). First maxilliped covered by an embryonic cuticle; coxa bore three setae, the basis eight setae; endopod 3‑segmented with 3, 2, and 3 setae proximally; exopod carried four terminal setae (Fig 4I). Second and third maxillipeds also covered by embryonic cuticle; coxa and basis without seta; endopods 5‑segmented; exopods bore six setae (Fig 5A and 5B). First pereopod covered by an embryonic cuticle; coxa and basis without seta; endopod possessed six setae; exopod bore six setae (Fig 5C). Telson with the posterior margin exhibited seven pairs of processes, an outermost spiniform pair, followed by five pairs of long plumose setae, and an innermost pair of short setae. As in *Spongicola venustus*, the first to sixth pairs enveloped within a hollow plumose embryonic cuticle (Fig 4J).

**Fig 4:**
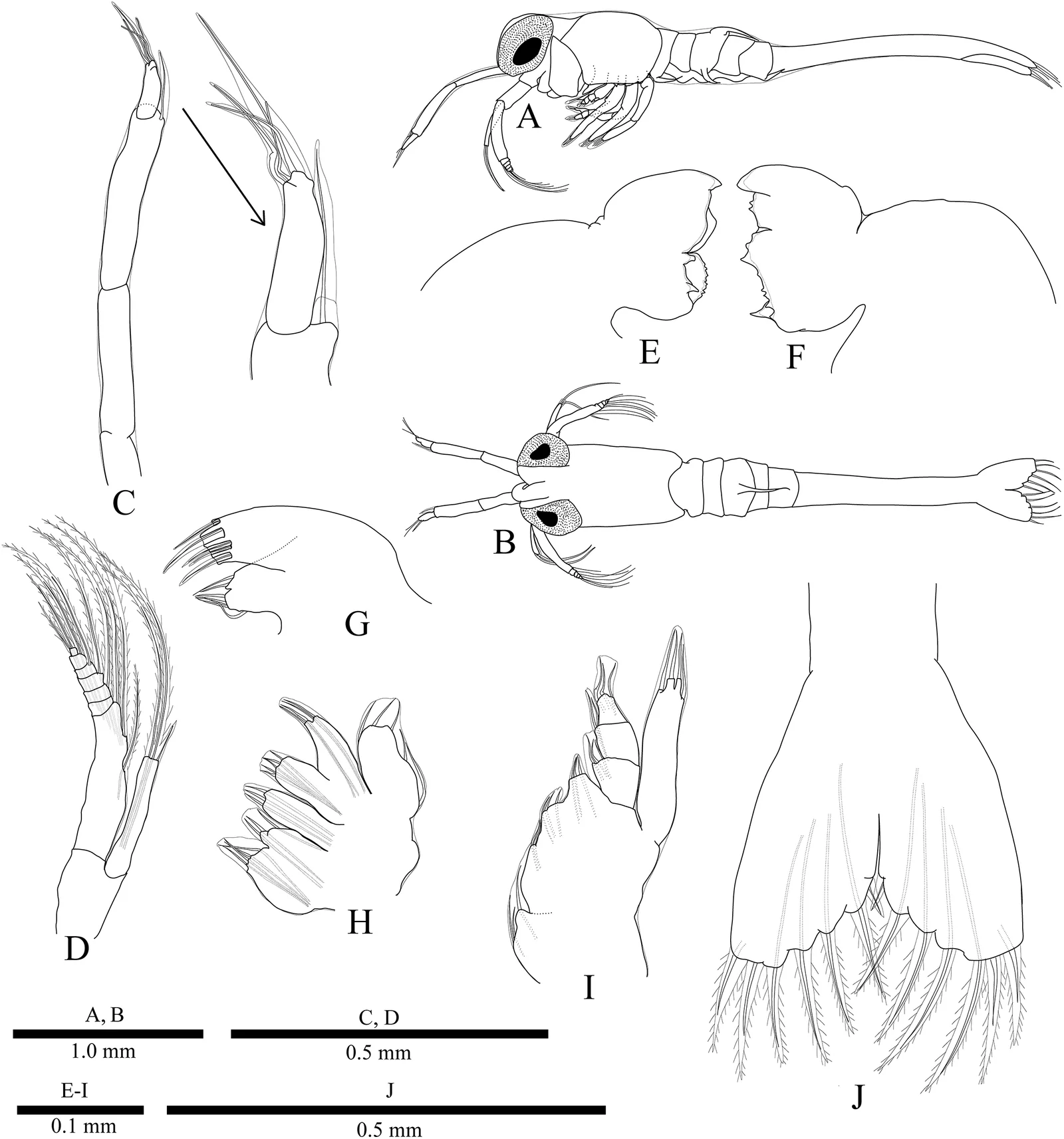
Prezoea of *Stenopus hispidus* (Olivier, 1811). A: whole animal, lateral view; B: same, dorsal view; C: antennule; D: antenna; E: right mandible, posteroventral view; F: left mandible, posteroventral view; G: maxillule; H: maxilla; I: 1st maxilliped; J: telson.

**Fig 5:**
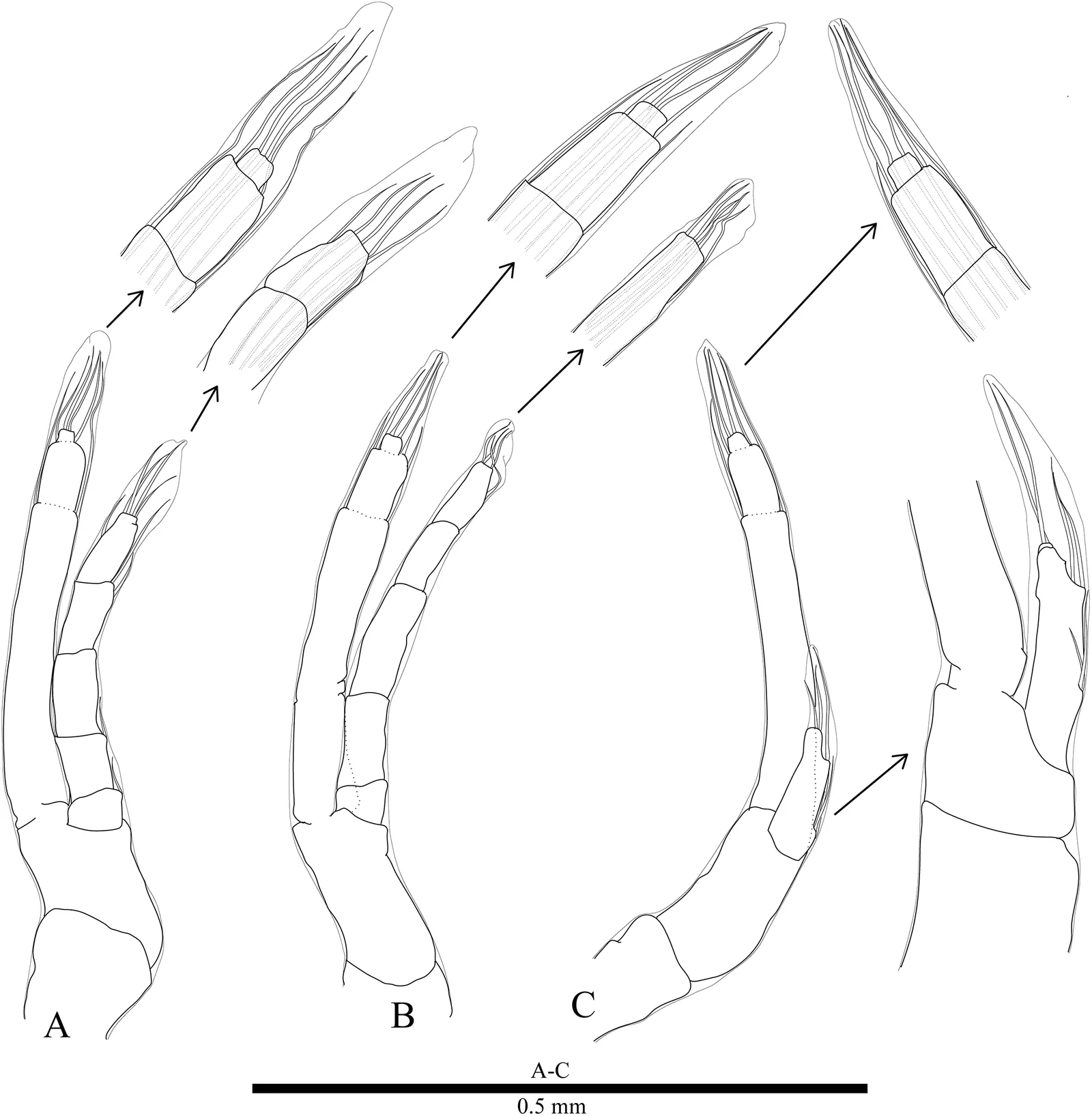
Prezoea of *Stenopus hispidus* (Olivier, 1811). A, B: 2nd & 3rd maxillipeds; C: 1st pereopod.

### 3. Observation of swimming behavior

Fig 6 shows frames from S3 and S4 Movies recording the swimming behavior of *Spongicola venustus* (A-1 to A-5) and *Stenopus hispidus* (B-1 to B-5), respectively. The prezoeae of both species swam primarily via rhythmic strokes of the antennal scales (scaphocerites), alternating between power and recovery phases. The antennal exopods moved posteriorly during the power phase, displacing water backward and generating forward propulsion (A-1 to A-3; B-1 to B-3), and returned to their initial position during the recovery phase (A-3 to A-5; B-3 to B-5). Stroke amplitude was relatively small, and the appendages remained rigid throughout both phases. Occasional rapid pleonal flexions provided supplementary thrust, resulting in brief accelerations or changes in orientation. This swimming mode contrasts sharply with that of the first zoeal stage, which relies on rhythmic beating of thoracic natatory appendages to achieve sustained telson-first swimming. In contrast, prezoeae swim cephalothorax-first using antennal propulsion while the thoracic appendages remain enclosed within the embryonic cuticle. Antenna-based propulsion in the examined prezoeae persisted until metamorphosis into the first zoeae.

**Fig 6:**
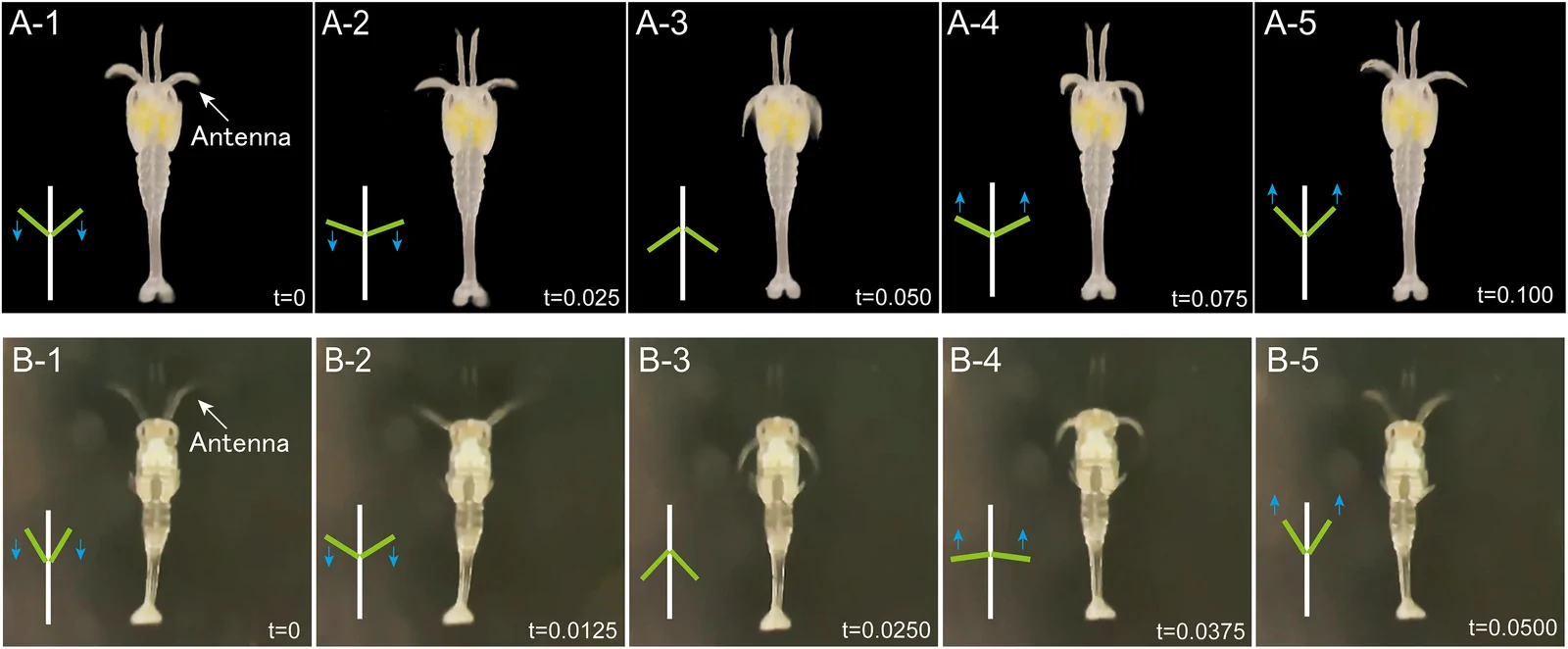
Ventral views of *Spongicola venustus* (De Haan, 1844) and *Stenopus hispidus* (Olivier, 1811) prezoeae showing their swimming behaviors. A-1–5: Frames of *Spongicola venustus* at 0.025‑s intervals (40 fps playback). B-1–5: Frames of *Stenopus hispidus* shown at 0.0125‑s intervals (80 fps playback). The white line denotes the body axis. The green lines indicate the antennae and are drawn symmetrically based on the angle of the right antenna measured from its basal region toward the midline. Arrows indicate the direction of antennal movement.

## Discussion

### Assessment of prezoeal normality and morphological characteristics

To evaluate our initial hypothesis that the prezoeal stage represents a normal and functionally specialized phase of the life cycle rather than an artificial or abnormal hatching event, we examined the morphology, behavior, and molting success of prezoeae in *Spongicola venustus* and *Stenopus hispidus*. In both species, all larvae consistently hatched while enclosed within a well-defined embryonic cuticle, actively swam immediately after hatching, and successfully molted into the first zoeae under suitable conditions. These observations support our hypothesis and demonstrate that the prezoeal stage in Stenopodidea is a regular developmental phase rather than an artifact of laboratory conditions or incomplete hatching. Furthermore, the occurrence of the same developmental pattern in both a deep-sea sponge-associated species and a shallow-water free-living species supports our hypothesis that prezoeal morphology and behavior are largely conserved despite substantial ecological differences between the two species. These findings suggest that the prezoeal stage represents an evolutionarily conserved strategy rather than a habitat-specific adaptation.

The combination of a compact morphology, functional swimming behavior, and rapid transition to the first zoeal stage further indicates that the prezoeal stage is more than a passive intermediate enclosed within an embryonic cuticle. Instead, it appears to represent a specialized transitional phase that facilitates successful emergence from the parental environment and promotes initial locomotion immediately after hatching. Such functions may be particularly important during the vulnerable period before the zoeal swimming appendages become fully functional.

The prezoeae of both stenopodidean species shared a distinctive suite of morphological characteristics, including a folded rostrum, ventrally folded pleonal processes, enlarged antennae bearing elongated plumose projections, and a telson equipped with conspicuous cuticular processes. These features contribute to a compact body configuration immediately after hatching while maintaining structures necessary for locomotion and subsequent development.

Compared with other decapod infraorders, stenopodidean prezoeae exhibit a unique pattern in the composition and plumosity of cuticular projections on the antennae and telson. Previous studies have shown that caridean prezoeae generally possess short antennules and antennae with naked setae and a telson bearing six pairs of short projections [4,15]. In anomurans, telsonal projections often bear thick conical accessory processes interpreted as a primitive setal condition [4,16–18], whereas brachyuran prezoeae typically possess a bifurcated telson with seven pairs of projections, although these may be reduced or absent in some taxa [4,19–21].

In contrast, both *Spongicola venustus* and *Stenopus hispidus* possess nine elongated projections on the antennal exopod, two on the endopod (with an additional small basal projection in *Stenopus hispidus*), and a medially divided telson bearing seven long projections and a pair of short median projections. Importantly, these projections are not merely cuticular extensions but function as sheaths enclosing the developing zoeal setae; these setae and their associated plumose accessory processes are visible within the sheaths. These morphological characteristics are consistent with previous descriptions of stenopodidean prezoeae [10,11] and clearly distinguish them from the prezoeae reported in other decapod infraorders.

In anomuran and brachyuran decapods, the prezoeae bear plumose processes not only on the telson but also on the first and second antennae. However, unlike those observed in stenopodidean shrimps, these processes do not fully enclose the developing zoeal setae, or in some cases consist solely of a cuticular membrane without any incorporated setae. The interiors of these processes are filled with prezoeal fluid which, as suggested by scanning electron microscopy observations, is thought to function as a hydraulic skeleton, thereby maintaining process shape and mechanical rigidity [2]. By contrast, the complete enclosure of developing zoeal setae within cuticular sheaths in stenopodidean prezoeae suggests a different structural strategy. This arrangement may allow elongate locomotory elements to remain protected and compact during emergence while preserving their rapid deployment following metamorphosis to the first zoeae.

### Swimming behavior

The prezoeae of stenopodidean shrimps observed in this study occasionally exhibited swimming driven by pleonal flexion, as reported for anomuran and brachyuran prezoeae. However, the predominant mode of locomotion was rhythmic movement of the second antennae. Previous research has reported that the prezoea of *Stenopus hispidus* swims using greatly enlarged antennae bearing long setae [12]. More recent studies have likewise reported that the prezoeae of stenopodidean shrimps rely primarily on the second antennae for swimming [10,11,13]. The present study corroborates these earlier descriptions based on video analysis, providing direct visual evidence that antennal swimming is a characteristic and predominant behavioral trait of the prezoeal stage in Stenopodidea. Furthermore, the occurrence of this same swimming mechanism in both a shallow-water free-living species (*Stenopus hispidus*) and a deep-sea sponge-associated species (*Spongicola venustus*) strongly indicates that antennal propulsion is an evolutionarily conserved feature across stenopodidean prezoeae. This finding supports the hypothesis that the prezoeal stage represents a functional and phylogenetically conserved strategy facilitating early post-hatching locomotion.

Reports of swimming behavior during the prezoeal stage in crustaceans are generally limited; however, studies on anomurans and brachyurans have shown that prezoeae do not swim using the maxillipeds, as zoeae do. Instead, locomotion is achieved through intermittent movements produced by pleonal flexion, often described as jerking behavior [17,20,22–24].

Prezoeae have been reported to swim by synchronous spasmodic movements of the antennules, antennae, and telson, and it has been suggested that the processes extending from these structures are involved in propulsion as well [4]. In addition, plumose setae on both the antennules and antennae of anomuran prezoeae have been suggested to generate thrust and enable swimming activity in concert with movements of the pleon and telson [18]. Taken together, these observations suggest that plumose processes function as oars that generate propulsive force, analogous to the natatory setae on the exopods of zoeal maxillipeds. This interpretation supports the view that the prezoea is not merely a larval form passively enclosed within a membrane, but instead possesses a relatively well-developed morphology equipped with setae and cuticular appendages specifically adapted to the characteristic swimming behavior observed during the prezoeal stage.

In the two stenopodidean species examined in this study, the prezoeae lack the well-developed cuticular processes on the antennules that are commonly observed in anomuran and brachyuran prezoeae. Instead, the antennules extend straight anteriorly from the cephalothorax and appear to be immobile. In contrast, the antennae bear a greater number of elongated processes compared with those of anomuran and brachyuran prezoeae. In addition, the antennal scale is relatively long and flexible, exhibiting considerable mobility. These morphological features indicate that stenopodidean prezoeae are structurally specialized for swimming via movements of the second antennae, which enables oriented locomotion with the cephalothorax directed toward the direction of movement. Because the thoracic natatory appendages remain enclosed within the embryonic cuticle at hatching, propulsion by the enlarged antennal exopods provides an effective temporary locomotor mechanism until the first zoeal swimming apparatus becomes fully functional. This locomotory system differs fundamentally from that of the first zoea, which depends on thoracic natatory appendages for sustained swimming. The transition from antennal propulsion in the prezoea to thoracic-appendage propulsion in the first zoea therefore represents a major functional shift in locomotory strategy during early development.

A comparable locomotion strategy has been reported for spiny lobster (Achelata), which hatches as a phyllosoma larva. Immediately after hatching, the larvae of *Palinurus* species exhibit a morphology resembling that of a nauplius larva, a stage designated as the “naupliosoma” [25]. The naupliosoma is characterized by a rounded body shape resulting from strong flexion and overlap of the cephalic and thoracic regions. In contrast, the pleon is short, underdeveloped, and enclosed within a cuticular covering. At the naupliosomal stage, the thoracic appendages which later serve as the primary swimming organs in the phyllosoma are folded against the body, with natatory setae remaining undeveloped, and the fourth and fifth pereopods present only as rudiments. In contrast, the second antennae are conspicuously developed and biramous, with the exopod bearing broad, plate-like swimming blades equipped with long plumose setae. It has been suggested that the naupliosoma uses vigorous movements of these highly developed second antennae to rapidly ascend to the water surface immediately after hatching. Because its mouthparts are not yet functional, the naupliosoma does not feed and undergoes molting to the subsequent phyllosomal stage within 4–6 h after hatching [25–27]. The reliance for locomotion on prominently developed antennae bearing swimming setae is a feature that naupliosomal larvae share with the prezoeae of stenopodidean shrimps. This similarity suggests that comparable adaptive selective pressures associated with successful emergence and early dispersal may have favored the independent evolution of these transient post-hatching stages, although the developmental stages themselves are not homologous.

### Duration of the prezoeal stage and its dependence on water temperature

In the present study, the duration of the prezoeal stage in both *Spongicola venustus* and *Stenopus hispidus* exhibited a strong inverse dependence on water temperature. For both species, even a 5°C increase in water temperature resulted in a more than two-fold reduction in prezoeal duration. Prezoeae maintained under warmer conditions completed metamorphosis to the first zoeal stage significantly faster than those reared at lower temperatures. These quantitative trends demonstrate that the duration of the prezoeal stage in stenopodidean shrimps is highly temperature-dependent. Because prezoeae employ a locomotory system that differs substantially from that of the subsequent zoeal stage, such thermal variation in prezoeal duration directly alters the time window during which larvae rely on antennal propulsion immediately after hatching. Consequently, water temperature regulates not only the overall developmental rate, but also the persistence of this specialized functional stage.

Comparable temperature-dependent variation in prezoeal duration has been documented in a wide range of decapod crustaceans. In some species, the embryonic cuticle is shed almost immediately upon hatching, whereas in many others the prezoeal stage persists for several minutes up to a few hours before molting to the first zoeal stage [4,17–20,22,28–33]. Substantial variation in the duration of the prezoeal stage has also been reported in astacideans. In the abbreviated-development species *Enoplometopus occidentalis*, the prezoeal stage can last from several hours up to approximately 12 h after hatching [29]. In contrast, in the direct-developing species *Metanephrops japonicus*, the first individuals begin to molt to the megalopa stage approximately 1 h after hatching, while it might take up to 22 h for all individuals within the same clutch to complete metamorphosis [34]. These studies illustrate that the prezoeal stage can vary considerably even among individuals within a single clutch.

The only comparable report currently available from Stenopodidea concerns *Microprosthema semilaeve*, in which prezoeae were observed to molt to the first zoeal stage within at least 6 h after hatching [13]. However, successful metamorphosis was recorded in only two of the 43 individuals examined. Given this extremely low success rate, it is possible that the reported duration reflected abnormal hatching conditions rather than the typical developmental pattern of the species. In contrast, the high metamorphic success observed in both species examined in the present study indicates that the durations recorded here represent normal developmental trajectories in stenopodidean shrimps under suitable conditions.

Temperature-dependent variation in the duration of the prezoeal stage has also been demonstrated in the naupliosomal larvae of achelatan lobsters. For instance, an experimental study on the effect of water temperature on *Jasus lalandii* [27] reported that 66% and 75% of naupliosomal larvae successfully molted to the first phyllosoma stage when they were maintained for 12 h at 5.5°C and for 15 min at 20°C, respectively. These findings provide clear evidence that the duration of transient post-hatching stages is strongly influenced by temperature across diverse decapod lineages. Together with the present results, they suggest that thermal regulation of developmental transitions may represent a widespread characteristic of short-lived post-hatching stages in decapod crustaceans.

The mechanism through which the prezoea sheds the embryonic cuticle and transitions to the first zoeal stage remains unresolved. However, this process closely resembles molting, a phenomenon commonly observed across all crustaceans. Water temperature has been shown to significantly influence molting intervals in crustaceans [35]. For example, in Decapoda, molting is generally regulated by a decrease in the levels of molt-inhibiting hormone, followed by the release of ecdysteroid molting hormones in response to this reduction [36–38]. Elevated water temperatures may enhance metabolic activity and enzyme reaction rates associated with hormone synthesis and regulation, thus potentially accelerating the hormonal cascade controlling cuticle shedding and shortening the time required for prezoeae to shed the embryonic cuticle and complete metamorphosis into the first zoeal stage.

### Biological significance of the prezoeal stage and its similarity to the pronymph stage in insects

Taken together, the morphological, behavioral, and developmental evidence obtained in the present study indicates that the prezoeal stage contributes to successful emergence, early locomotion, and initial dispersal immediately after hatching. In stenopodidean shrimps, the first zoea possesses a long rostrum, conspicuous spines, and well-developed appendages that would likely hinder passage through confined spaces immediately after hatching. By contrast, these structures remain folded beneath the embryonic cuticle during the prezoeal stage, resulting in a compact body form that may facilitate emergence from the brood mass while reducing the risk of entanglement with egg stalks, attached eggs, and the dense setation of the maternal pleopods [23].

This interpretation may be particularly relevant for symbiotic taxa. Prezoeae of the porcellanid crab *Polyonyx gibbesi*, which lives commensally within the narrow tubes of polychaete worms, have been reported to actively escape from the host tube immediately after hatching [39]. The zoeae of porcellanid crabs possess extremely elongated spines, and emergence through the confined space of the host tube would therefore involve a substantial risk of entanglement. Hatching as a compact prezoea is likely to facilitate successful escape from this structurally restrictive environment. A similar advantage may occur in *Spongicola venustus*, whose larvae must leave the atrial cavity of a glass sponge through a complex mesh-like skeletal framework. In such situations, a compact prezoeal morphology may facilitate successful emergence, whereas a fully expanded zoea bearing long spines and projections would be more susceptible to mechanical interference.

In addition to facilitating emergence, the prezoeae examined in the present study immediately initiated active swimming using the second antennae after hatching. Because the thoracic natatory appendages remain enclosed within the embryonic cuticle, antennal propulsion provides an effective temporary locomotor mechanism until the zoeal swimming apparatus becomes functional. Such swimming behavior may promote rapid ascent and initial dispersal during a particularly vulnerable period immediately following hatching. Together, these observations suggest that the prezoeal stage contributes both to successful emergence from the parental environment and to early post-hatching dispersal.

Based on observations of hatching behavior and morphological characteristics of the prezoea in *Erimacrus isenbeckii*, Sasaki and Mihara [20] proposed that this stage represents a developmental phase specifically adapted for embryonic escape and noted its resemblance to the pronymph stage observed in insects. The present findings provide additional support for this interpretation by demonstrating that stenopodidean prezoeae combine a compact morphology with active post-hatching locomotion.

A functional interpretation of the prezoea in stenopodidean shrimps therefore reveals intriguing parallels with the pronymph stage observed in several hemimetabolous insects [40–43]. However, it is important to emphasize that the prezoea and pronymph are not homologous developmental stages, and this comparison does not imply absolute functional equivalence. Pronymphs are short-lived post-hatching forms characterized by a flexible cuticle, folded appendages, and rapid transition to the subsequent developmental stage. They are generally interpreted as adaptations facilitating escape from the egg shell, oviposition site, or immediate post-hatching environment [42,43].

Despite their distinct phylogenetic origins and morphological structures, insect pronymphs and decapod prezoeae share several notable functional characteristics: (1) a compact body enclosed within a cuticle and containing folded appendages, (2) a very short duration, and (3) specialized movements that facilitate emergence and locomotion immediately after hatching. In hemimetabolous insects, such movements enable escape from soil, plant tissues, or the eggshell [42–45], whereas in stenopodidean shrimps they may facilitate emergence from brood masses or host structures and promote rapid ascent into the water column.

These similarities suggest that comparable ecological constraints, namely the need to overcome physical barriers and achieve effective locomotion immediately after hatching, can independently favor the evolution of analogous transient post-hatching forms across divergent arthropod lineages. Although pronymphs and prezoeae are not homologous developmental stages, the remarkable functional parallels between them highlight how similar selective pressures may repeatedly drive the evolution of compact, short-lived, and behaviorally specialized stages that enhance survival during one of the most vulnerable periods of the life cycle.

## Supporting information

S1 Movie Video recording made with a Nikon D5600 camera showing the transformation from prezoea to first zoea in *Spongicola venustus.* The rostrum extends while pleonal movements pull the embryonic cuticle away from the cephalothorax and its appendages via the connecting membrane. Subsequently, while gently moving the appendages in an apparent acclimatory manner, vigorous pleonal movements enable the shedding of the embryonic cuticle covering the telson, thereby completing the transformation into the first zoea.
(MP4)

S2 Movie Video recording made with a Nikon D5600 camera showing the swimming behavior of a *Stenopus hispidus* larva and its transformation from prezoea to first zoea. The larva swims freely using the antennae. Subsequently, as observed in S1 Movie for *Spongicola venustus*, the transformation to the first zoea is completed.
(MP4)

S3 Movie Video recording showing the swimming behavior of *Spongicola venustus.* Recorded from an 8 × slow-motion movie using an iPhone 11.
(MP4)

S4 Movie Video recording showing the swimming behavior of *Stenopus hispidus.* Recorded from an 8 × slow-motion movie using an iPhone 15.
(MP4)
