# Supplementary material for: Morphology and swimming behavior of prezoeae in two stenopodidean shrimps: Functional implications for early larval life
Source: PLoS One. 2026 Nov 10;21(11):e0361633. doi: 10.1371/journal.pone.0361633 (PMC13750586; doi:10.1371/journal.pone.0361633)

PLOS One

Morphology and swimming behavior of prezoeae in two stenopodidean shrimps: functional implications for early larval life

--Manuscript Draft--

Manuscript Number:	PONE-D-26-25140
Article Type:	Research Article
Full Title:	Morphology and swimming behavior of prezoeae in two stenopodidean shrimps: functional implications for early larval life
Short Title:	Prezoeal morphology and swimming in stenopodidean shrimps
Corresponding Author:	Mikihiro Nakagawa, MSc<br>Kochi University: Kochi Daigaku<br>Tosa, Kochi JAPAN
Keywords:	larvae; prezoea; stenopodidea; Spongicola venustus; Stenopus hispidus; decapod crustacean; larval development; larval morphology; larval behavior
Abstract:	Most decapod crustaceans undergo a brief prezoeal stage immediately after hatching, during which the body remains enclosed within a thin embryonic cuticle. Although this stage typically lasts only minutes and is often omitted from larval descriptions, detailed morphological records exist for various taxa. However, these descriptions remain fragmentary, and studies linking morphology to behavior are almost nonexistent, leaving the biological and evolutionary significance of this short developmental stage largely unexplored. This study documents the morphology and swimming behavior of two stenopodidean shrimps, i.e., Spongicola venustus (De Haan, 1844), a deep-sea sponge-associate, and Stenopus hispidus (Olivier, 1811), a shallow-water free-living species, during the prezoeal stage. The aim was to clarify phylogenetically conserved traits and the biological significance of morphology and behavior in relation to ecological type. Ovigerous females were collected from Tosa Bay, Japan, and reared in aquaria until hatching. Morphological observations were conducted on preserved specimens, whereas swimming behavior was analyzed using slow-motion microscopy movies. Both species hatched in the prezoeal stage, enclosed in an embryonic cuticle as observed in other taxa, with key zoeal features visible beneath. The prezoeae swam via rhythmic strokes of the antennae (scaphocerites), occasionally aided by abdominal flexions. Plumose setae on the antennae and telson appeared to enhance propulsion. These traits may reduce the risk of snagging by allowing elongated appendages to remain folded and may facilitate emergence from egg chambers or sponge mesh. They are also likely to be crucial for promoting initial ascent immediately after hatching. This swimming activity driven by cephalic appendages in prezoeae resembles naupliar locomotion, suggesting partial retention of primitive locomotor patterns. In addition, similar to insect pronymphs, the prezoeal stage may be effective in overcoming physical barriers associated with hatching, representing a response to shared selective pressures during early development. These findings provide new insights into the evolutionary diversity of developmental strategies in decapod crustaceans.
Order of Authors:	Mikihiro Nakagawa, MSc
	Tomomi Saito, phD
Additional Information:	
**Question**	**Response**
**Financial Disclosure**<br><br>Enter a financial disclosure statement that describes the sources of funding for the work included in this submission. Review the submission guidelines for detailed requirements. View published research articles from *PLOS ONE* for specific	Yes

Powered by Editorial Manager® and ProduXion Manager® from Aries Systems Corporation

examples.<br><br>This statement is required for submission and **will appear in the published article** if the submission is accepted. Please make sure it is accurate.<br><br>**Funded studies**<br>Enter a statement with the following details:<br>• Initials of the authors who received each award<br>• Grant numbers awarded to each author<br>• The full name of each funder<br>• URL of each funder website<br>• Did the sponsors or funders play any role in the study design, data collection and analysis, decision to publish, or preparation of the manuscript?<br><br>Did you receive funding for this work?	
Please add funding details.<br>as follow-up to "**Financial Disclosure**<br><br>Enter a financial disclosure statement that describes the sources of funding for the work included in this submission. Review the submission guidelines for detailed requirements. View published research articles from *PLOS ONE* for specific examples.<br><br>This statement is required for submission and **will appear in the published article** if the submission is accepted. Please make sure it is accurate.<br><br>**Funded studies**<br>Enter a statement with the following details:<br>• Initials of the authors who received each award<br>• Grant numbers awarded to each author<br>• The full name of each funder<br>• URL of each funder website<br>• Did the sponsors or funders play any role in the study design, data collection and analysis, decision to publish, or preparation of the manuscript?	This study was supported by the Kuroshio Biological Research Institute Foundation (Research Grant Program 2024) and JST SPRING (Grant Number JPMJSP2166) to MN. The funders had no role in study design, data collection and analysis, decision to publish, or preparation of the manuscript.

Powered by Editorial Manager® and ProduXion Manager® from Aries Systems Corporation

Did you receive funding for this work?"	
Please select the country of your main research funder (please select carefully as in some cases this is used in fee calculation).<br>as follow-up to "**Financial Disclosure**<br><br>Enter a financial disclosure statement that describes the sources of funding for the work included in this submission. Review the submission guidelines for detailed requirements. View published research articles from *PLOS ONE* for specific examples.<br><br>This statement is required for submission and **will appear in the published article** if the submission is accepted. Please make sure it is accurate.<br><br>**Funded studies**<br>Enter a statement with the following details:<br>• Initials of the authors who received each award<br>• Grant numbers awarded to each author<br>• The full name of each funder<br>• URL of each funder website<br>• Did the sponsors or funders play any role in the study design, data collection and analysis, decision to publish, or preparation of the manuscript?<br><br>Did you receive funding for this work?"	JAPAN - JP
**Competing Interests**<br><br>Use the instructions below to enter a competing interest statement for this submission. On behalf of all authors, disclose any competing interests that could be perceived to bias this work—acknowledging all financial support and any other relevant financial or non-financial competing interests.	The authors have declared that no competing interests exist.

Powered by Editorial Manager® and ProduXion Manager® from Aries Systems Corporation

This statement is required for submission and **will appear in the published article** if the submission is accepted. Please make sure it is accurate and that any funding sources listed in your Funding Information later in the submission form are also declared in your Financial Disclosure statement.<br><br>View published research articles from *PLOS ONE* for specific examples.<br><br>**NO authors have competing interests**<br><br>Enter: *The authors have declared that no competing interests exist.*<br><br>**Authors with competing interests**<br><br>Enter competing interest details beginning with this statement:<br><br>*I have read the journal's policy and the authors of this manuscript have the following competing interests: [insert competing interests here]*<br><br>* typeset	
**Ethics Statement**<br><br>Enter an ethics statement for this submission. This statement is required if the study involved:<br><br>• Human participants<br>• Human specimens or tissue<br>• Vertebrate animals or cephalopods<br>• Vertebrate embryos or tissues<br>• Field research<br><br>Write "N/A" if the submission does not require an ethics statement.<br><br>General guidance is provided below. Consult the submission guidelines for detailed instructions. **Make sure that all information entered here is included in the Methods section of the manuscript.**	N/A

**Format for specific study types**<br><br>**Human Subject Research (involving human participants and/or tissue)**<br>• Give the name of the institutional review board or ethics committee that approved the study<br>• Include the approval number and/or a statement indicating approval of this research<br>• Indicate the form of consent obtained (written/oral) or the reason that consent was not obtained (e.g. the data were analyzed anonymously)<br><br>**Animal Research (involving vertebrate animals, embryos or tissues)**<br>• Provide the name of the Institutional Animal Care and Use Committee (IACUC) or other relevant ethics board that reviewed the study protocol, and indicate whether they approved this research or granted a formal waiver of ethical approval<br>• Include an approval number if one was obtained<br>• If the study involved *non-human primates*, add *additional details* about animal welfare and steps taken to ameliorate suffering<br>• If anesthesia, euthanasia, or any kind of animal sacrifice is part of the study, include briefly which substances and/or methods were applied<br><br>**Field Research**<br><br>Include the following details if this study involves the collection of plant, animal, or other materials from a natural setting:<br>• Field permit number<br>• Name of the institution or relevant body that granted permission	
**Data Availability**<br><br>Before publication, Authors are required to make fully available and without restriction all data underlying their findings. Sharing the data underlying your findings increases the visibility, credibility, and impact of your work, while supporting transparency and future scientific	All relevant data are within the manuscript and its Supporting Information files. Supplementary movies (S1–S4) are available via Figshare at the following private link for peer review: https://figshare.com/s/7657c0198fef7ccaf70e. These data will be made publicly available with a DOI upon acceptance.

Powered by Editorial Manager® and ProduXion Manager® from Aries Systems Corporation

discovery. Please see our PLOS Data Policy page for detailed information on this policy.<br><br>A **Data Availability Statement**, detailing where the data can be accessed, is required at first submission. Insert your Data Availability Statement in the box below.<br><br>Please see the data reporting section of our submission guidelines for instructions on what you need to include in your Data Availability Statement. Examples of acceptable Data Availability Statements are included here for reference:<br>1. The minimal data set [and accompanying code, where generated] is available at [repository name] via [DOI hyperlink]<br>2. No data was generated by this study. The following existing data sources were used: [dataset name] from [dataset location] available via [web address or DOI hyperlink].<br>3. For Study Protocols: No datasets were generated or analysed during the current study. All relevant data from this study will be made available upon study completion.<br><br>*PLOS allows rare exemptions to address legal and ethical concerns. If you have legal or ethical restrictions, please use the box below to detail these in full sentences for the Journal team to consider.*<br><br>This statement will be typeset if the manuscript is accepted for publication.	

Powered by Editorial Manager® and ProduXion Manager® from Aries Systems Corporation

Manuscript
Click here to access/download;Manuscript;ForSubmit_Nakagawa and

Morphology and swimming behavior of prezoeae in two stenopodidean shrimps: functional implications for early larval life

Mikihiro Nakagawa[1]*, Tomomi Saito[1]¶

[1] Usa Marine Biological Institute, Kochi University, Kochi, Japan

* Corresponding author

E-mail: nmikinmiki2000@gmail.com (MN)

Abstract

Most decapod crustaceans undergo a brief prezoeal stage immediately after hatching, during which the body remains enclosed within a thin embryonic cuticle. Although this stage typically lasts only minutes and is often omitted from larval descriptions, detailed morphological records exist for various taxa. However, these descriptions remain fragmentary, and studies linking morphology to behavior are almost nonexistent, leaving the biological and evolutionary significance of this short developmental stage largely unexplored. This study documents the morphology and swimming behavior of two stenopodidean shrimps, i.e., *Spongicola venustus* (De Haan, 1844), a deep-sea sponge-associate, and *Stenopus hispidus* (Olivier, 1811), a shallow-water free-living species, during the prezoeal stage. The aim was to clarify phylogenetically conserved traits and the biological significance of morphology and behavior in relation to ecological type. Ovigerous females were collected from Tosa Bay, Japan, and reared in aquaria until hatching. Morphological observations were conducted on preserved specimens, whereas swimming behavior was analyzed using slow-motion microscopy movies. Both species hatched in the prezoeal stage, enclosed in an embryonic cuticle as observed in other taxa, with key zoeal features visible beneath. The prezoeae swam via rhythmic strokes of the antennae (scaphocerites), occasionally aided by abdominal flexions. Plumose setae on the antennae and telson appeared to enhance propulsion. These traits may reduce the risk of snagging by allowing elongated appendages to remain folded and may facilitate emergence from egg

chambers or sponge mesh. They are also likely to be crucial for promoting initial ascent immediately after hatching. This swimming activity driven by cephalic appendages in prezoeae resembles naupliar locomotion, suggesting partial retention of primitive locomotor patterns. In addition, similar to insect pronymphs, the prezoeal stage may be effective in overcoming physical barriers associated with hatching, representing a response to shared selective pressures during early development. These findings provide new insights into the evolutionary diversity of developmental strategies in decapod crustaceans.

Introduction

The prezoeal stage observed in decapod crustaceans (Pleocyemata) is defined as either the final stage of embryonic development or the first larval form immediately after hatching [1]. Morphologically, the entire body is enclosed within a thin membrane known as the embryonic cuticle. This stage lasts for an extremely short time, ranging from a few minutes to several hours, and cases have been reported in which prezoeae fail to molt and die following abnormal hatching under certain conditions, such as reduced salinity [2]. Consequently, whether the prezoea should be regarded as a true larval stage has long been a subject of debate [2, 3]. The accumulation of detailed morphological descriptions, particularly for brachyuran crabs, in recent years has led to increasing recognition of the prezoeal stage as a regular phase of the life cycle [4, 5]. Nevertheless, some

review articles continue to describe it merely as the terminal embryonic stage [6], which indicates that its biological interpretation remains incomplete. The most critical issue in understanding the nature of the prezoea lies in elucidating its biological significance; however, studies explicitly addressing this question are extremely limited. Addressing this knowledge gap requires comparative and generalized analyses of prezoeal morphology across a wide range of decapod taxa. At present, available knowledge is strongly biased toward brachyurans, with information on carideans and other decapod groups remaining scarce.

In stenopodidean shrimps, the prezoeal stage was first described in members of the family Spongicolidae examined during the Challenger expedition (1872-1876) [7, 8]. At that time, this stage was referred to by the unique term “brephalos”; however, subsequent reviews suggested that it corresponded to the prezoeal stage as currently defined [9–11]. Thereafter, fragmentary morphological observations of the prezoea have been reported for *Stenopus hispidus* [12] and *Microprosthema semilaeve* [13], including features such as a folded rostrum, well-developed second antennae, and poorly developed setation on the appendages. In addition, the direct-developing species *Spongiocaris japonica* has been shown to hatch as a prezoea-like form enclosed within an embryonic cuticle [14]. Taken together, these observations suggest that the prezoeal stage is widely present across Stenopodidea [10, 11]. Nevertheless, detailed morphological analyses based on dissection, quantitative evaluation of swimming behavior via video analysis, and comprehensive

descriptions of the metamorphic transition to the first zoeal stage remain extremely limited.

Stenopodidea is a decapod lineage that has evolved diverse ecological adaptations, with organisms being able to dwell in different environments ranging from shallow to deep waters and adopt different lifestyles, from free living to symbiosis. Understanding early life-history stages is therefore essential for elucidating their adaptive evolution and patterns of pelagic dispersal. In this study, we conducted a systematic comparative analysis of prezoeal morphology and swimming behavior in two stenopodidean shrimps: the deep-sea, sponge-associated *Spongicola venustus* and the shallow-water, free-living *St. hispidus*. In addition, we report the temperature dependence of metamorphic timing on the prezoeal stage. Through detailed observations of stenopodidean prezoeae, this study re-evaluates the biological significance of the prezoeal stage from the perspectives of evolutionary developmental biology and comparative behavioral biology, moving beyond the previously held view of a collection of isolated phenomena. This approach provides insight into survival strategies operating immediately after hatching in decapod crustaceans.

Materials and Methods

Collection of ovigerous shrimps

Ovigerous females of ~~Sp. venustus~~ were collected from glass sponges caught during two research

cruises conducted in Tosa Bay, Japan. Sampling was carried out by the training ship "Toyohata-maru" in March 2023 using a trawl net (mouth width: 1.5 m; mesh size: 2.0 mm), and by the bottom trawling vessel "Tsukasa-maru" in February 2024 using a bottom trawl net. Ovigerous females of ~~*St. hispidus*~~ were collected from a fishing port quay and rocky reefs using dip nets or by scuba diving between October 2022 and July 2025 in Tosa City and Otsuki Town, Hata District, Kochi Prefecture, Japan. The specimens of both species were maintained in rearing facilities at the Usa Marine Biological Institute, Kochi University. These shrimp species are not listed under any conservation laws, and collection was conducted under permission from Kochi Prefecture and relevant local fishery cooperatives.

Adult rearing conditions

Adults of *Sp. venustus* were maintained in a small tank (15.5 × 14.0 × 11.5 cm) placed within a 120-cm overflow aquarium under dark conditions (achieved via blackout curtains). Water temperature was maintained at 15°C–19°C, salinity at 28–34 ppt, and pH at 8.1–8.2. Adults of *St. hispidus* were maintained in a standard aquarium tank (size of approximately 120 × 45 × 45 cm) at a water temperature of 24°C–26°C, salinity of 28–34 ppt, and pH 8.1–8.3. Lighting followed the ambient L:D cycle in the laboratory until prezoeae were released. Both species were fed a commercial crustacean feed paste (Itosui Ebi no Shushoku Nattokin).

Morphological and behavioral observations

To evaluate temperature-dependent metamorphic rates, we measured the duration from hatching to complete transformation into the first zoea under two water temperatures for each species: 15°C and 20°C for *Sp. venustus*, and 20°C and 25°C for *St. hispidus*. Newly hatched larvae (*Sp. venustus*: November 2024; *St. hispidus*: June and July 2025) were fixed in sugar-formalin (20 g sucrose in 100 ml of 3% neutral buffered formalin). Body size was measured to the nearest 0.01 mm using an ocular micrometer under a stereomicroscope. Total length (TL) was defined as the linear distance from the anterior tip of the carapace along the midline to the posterior margin of the telson. Carapace length (CL) was defined as the linear distance between the anterior margin of the carapace at the base of the rostrum (excluding the folded rostrum) and the posterior margin of the carapace along the midline. Appendages were dissected under a stereomicroscope and sketched using a drawing device (Nikon Y-IDT) attached to a biological microscope (Nikon ECLIPSE E400). Shrimp morphology was sketched using a drawing tube (Nikon P-IDT) attached to a stereomicroscope (Nikon SMZ800). Lateral views and the metamorphic transition from the prezoeal to first zoeal stage were recorded using a digital camera (Nikon D5600) mounted on a stereomicroscope. Swimming behavior was recorded at 8× slow motion using a smartphone camera (iPhone 11 and 15) mounted on the stereomicroscope. Image sequences were extracted and processed using Adobe Premiere Pro and subsequently subjected to color correction and trimming in Adobe Photoshop.

Results

1. Hatching prezoeae and transformation to first zoeae

At the time of collection, the shrimp eggs contained the blue yolk characteristic of the early stages of embryogenesis. During the late embryonic stage, this blue yolk was progressively absorbed, and a yellow yolk became visible through the developing embryos, with the eyes clearly discernible immediately prior to hatching. Hatching typically started a few hours after sunset and continued over two consecutive nights. In both species, all embryos hatched as prezoeae from the egg cases attached to the female's pleopods (Fig. 1). Following hatching, the prezoeae immediately initiated upward swimming toward the surface (details of swimming behavior are described below) and ceased to do so after a short interval. Subsequently, the rostrum—initially folded beneath the carapace—began to extend, and the embryonic cuticle covering the cephalothorax, including appendages from the first antennule to the first pereopod, was gradually pulled away via the connecting membrane by extension movements of the abdomen. Once the rostrum was fully extended, the cephalothoracic embryonic cuticle, which had been drawn posteriorly by the telson, detached. Finally, vigorous abdominal flexions caused the embryonic cuticle surrounding the telson to separate, allowing the previously folded processes and setae beneath the cuticle to expand completely, thereby completing the transformation into the first zoea. The process of transformation from prezoea to first zoea is presented in Movie S1 for *Sp. venustus* and in Movie S2 for *St. hispidus.*

The time required from hatching to completion of the molt into the first zoea varied significantly with temperature. In *Sp. venustus*, the mean duration was 1802 ± 228 s at 15°C (n = 10) and 949 ± 209 s at 20°C (n = 20) ($P < 0.05$), whereas in *St. hispidus*, it was 887 ± 425 s at 20°C (n = 21) and 421 ± 150 s at 25°C (n = 24) ($P < 0.05$) (Fig. 2).

Fig. 1 Hatched prezoeae of *Spongicola venustus* (De Haan, 1844) (A) and *Stenopus hispidus* (Olivier, 1811) (B).

Fig. 2 Duration of the prezoeal stage in *Spongicola venustus* (De Haan, 1844) (A) and *Stenopus hispidus* (Olivier, 1811) (B)

2. Morphological observations

The entire shrimp body is enclosed within a thin embryonic cuticle. The carapace is smooth and lacks projections, with the rostrum folded beneath it. Abdominal processes are folded ventrally, and the tergum is smooth. The mandible is laterally constricted. The setae of the thoracic appendages are partly embedded in basal musculature and remain undeveloped within the cuticle, whereas those on the antennae and telson are enclosed in hollow cuticular sheaths. Some setae present in the first zoea are not exposed at this stage, likely due to immaturity or concealment; therefore, the following description is based only on observable structures.

Description of the prezoeal stage in *Spongicola venustus* (De Haan, 1844) (Fig 3)

Dimentions: CL = 0.92 ± 0.02 mm; TL = 3.41 ± 0.07 mm (n = 5). Carapace smooth, covered by a thin embryonic cuticle. Eyes sessile and pigmented. Rostrum folded beneath the carapace. Abdomen possessed protrusions on the posterior ventral margins of the first to fifth somites, folded ventrally (Fig 3A, A'). Antennule biramous and 2-segmented; endopod bore one long seta, whereas exopod carried two setae and three aesthetascs (Fig 3B). Antenna biramous and 2-segmented; endopod possessed two long plumose setae; exopod (scaphocerite) with an articulated terminal segment bearing nine long setae, each enclosed within a hollow plumose embryonic cuticle (Fig 3C). Mandibles asymmetrical, with well-developed incisor and molar processes (Fig 3D, E). Maxillule with a coxal endite bearing six setae and a basial endite bearing five setae, all enclosed within a hollow embryonic cuticle; endopod rudimentary (Fig 3F). Maxilla possessed a bilobed coxal endite with eight and three setae, and a bilobed basial endite with three and five setae; endopod with two setae; scaphognathite bore six marginal setae (Fig 3G). First maxilliped covered by an embryonic cuticle; coxa bore two setae, basis with eight setae; endopod three-segmented with 2, 2, and 4 setae proximally; exopod possessed four terminal setae, partially exposed (Fig 3H). Second and third maxillipeds also covered by embryonic cuticle; each endopod five-segmented; each exopod carried six setae (Fig 3I, J). First pereopod covered by an embryonic cuticle; endopod bore five setae;

exopod carried six setae (Fig 3K). Telson with posterior margin bore seven pairs of processes an outermost spiniform pair, followed by five pairs of long setae, and an innermost short pair, first to sixth pairs enclosed within a hollow plumose embryonic cuticle (Fig 3L).

Fig. 3 Prezoea of *Spongicola venustus* (De Haan, 1844). A: whole animal, lateral view; A': same, dorsal view; B: antennule; C: antenna; D, right mandible, posteroventral view; E, left mandible, posteroventral view; F: maxillule; G: maxilla; H–J: 1st–3rd maxillipeds; K: 1st pereopod; L: telson.

Description of prezoea in *Stenopus hispidus* (Olivier, 1811) (Figs 4, 5)

Dimensions: CL = 0.78 ± 0.02 mm; TL = 2.78 ± 0.08 mm (n = 5). Carapace smooth, covered by an embryonic cuticle, generally similar to that of *Sp. venustus*. Eyes sessile and pigmented. Abdomen possessed protrusions on the posterior ventral margins of the first and third somites, folded ventrally, and a dorsal spine on the third somite that remained folded beneath the embryonic cuticle (Fig 4A, A'). Antennule biramous and 2-segmented; endopod bore one long seta; exopod carried one seta and three aesthetascs (Fig 4B). Antenna biramous and 2-segmented; endopod possessed two long plumose setae and one short seta, whereas exopod (scaphocerite) with an articulated terminal segment bearing nine long setae, each encased within a hollow plumose embryonic cuticle (Fig 4C). Mandibles asymmetrical, with well-developed incisor and molar processes (Fig 4D, E). Maxillule

with a coxal endite bearing six setae and a basial endite bearing five setae, all enclosed within a hollow embryonic cuticle; endopod rudimentary (Fig 4F). Maxilla possessed a bilobed coxal endite with four and three setae, and a bilobed basial endite with three and five setae; endopod carried four setae; scaphognathite bore five marginal setae (Fig 4G). First maxilliped covered by an embryonic cuticle; coxa bore three setae, the basis eight setae; endopod three-segmented with 3, 2, and 3 setae proximally; exopod carried four terminal setae (Fig 5A). Second and third maxillipeds also covered by an embryonic cuticle; endopods five-segmented; exopods bore six setae (Fig 5A, B). First pereopod covered by an embryonic cuticle; endopod possessed six setae; exopod bore six setae (Fig 5C). Telson with the posterior margin exhibited seven pairs of processes, an outermost spiniform pair, followed by five pairs of long plumose setae, and an innermost pair of short setae. As in *Sp. venustus*, the first to sixth pairs enveloped within a hollow plumose embryonic cuticle (Fig 4I).

Fig. 4 Prezoea of *Stenopus hispidus* (Olivier, 1811). A: whole animal, lateral view; A’: same, dorsal view; B: antennule; C: antenna; D, right mandible, posteroventral view; E, left mandible, posteroventral view; F: maxillule; G: maxilla; H: 1st maxillipeds; I: telson.

Fig. 5 Prezoea of *Stenopus hispidus* (Olivier, 1811). A, B: 1st & 2nd maxillipeds; C: 1st pereopod.

3. Observation of swimming behavior

Figure 6 shows frames from Movie S3 and Movie S4 recording the swimming behavior of *Sp. venustus* (A1 to A5) and *St. hispidus* (B1 to B5), respectively. The prezoeae of both species swam primarily via rhythmic strokes of the antennal scales (scaphocerites), alternating between power and recovery phases. The antennal exopods moved posteriorly during the power phase, displacing water backward and generating forward propulsion (A1 to A3; B1 to B3), and returned to their initial position during the recovery phase (A3 to A5; B3 to B5). Stroke amplitude was relatively small, and the appendages remained rigid throughout both phases. Occasional rapid abdominal flexions provided supplementary thrust, resulting in brief accelerations or changes in orientation. This swimming mode contrasts sharply with that of the first zoea, which relies on natatory limbs for sustained telson-first swimming. Antenna-based propulsion in the examined prezoeae persisted until metamorphosis into the first zoeae.

Fig. 6 Ventral view of *Spongicola venustus* (De Haan, 1844) and *Stenopus hispidus* (Olivier, 1811) prezoeae showing their swimming behavior. A1–5: Frames of *Sp. venustus* at 0.025-s intervals (40 fps playback). B1–5: Frames of *St. hispidus* shown at 0.0125-s intervals (80 fps playback). The white line denotes the body axis. The green lines indicate the antennae and are drawn symmetrically based on the angle of the right antenna measured from its basal region toward the

midline. Arrows indicate the direction of antennal movement.

Discussion

Assessment of prezoeal normality and morphological characteristics

In this study, we confirmed that two shrimp species with markedly different habitats, *Sp. venustus* and *St. hispidus*, hatched while still enclosed within the prezoeal cuticle and swam functionally using the antennae. After a period of 207–2171 s following hatching, the larvae shed the prezoeal cuticle and metamorphosed normally into first zoeae. These observations indicate that stenopodidean shrimps hatch as prezoeae as part of their normal developmental process.

Previous studies have reported that the number and degree of plumosity of projections on the antennules (also known as first antennae), antennae, and telson of decapod prezoea vary among taxa [6]. In Caridea, although documented cases are limited, the first and second antennae are short and bear naked setae, and the telson possesses six pairs of short, naked projections [4, 15]. In Anomura, both sides of telsonal projections exhibit thick, conical accessory processes, which are considered as a more primitive version of setae [4, 16–18]. In Brachyura, the telson is generally bifurcated and bears seven pairs of cuticular projections; however, exceptions exist, including species in which

these projections are entirely undeveloped [4, 19–21].

The prezoeae of stenopodidean shrimps exhibit distinctive features in terms of both the composition and plumosity of cuticular projections on the antennae and telson, allowing them to be clearly distinguished from the prezoeae of other infraorders. In the two shrimp species examined, nine long projections are present on the antennal exopod, whereas the endopod bears two projections, with *St. hispidus* additionally possessing a small basal projection. The telson is smaller in the prezoea than in the first zoea, is divided medially into two lobes, and bears seven long projections together with a single pair of short median projections. In addition, these cuticular projections function as sheaths for the setae that develop in the zoeal stage. More specifically, the setae extend into the projections and are visible through the cuticle. Plumose accessory processes are arranged along the outer margins of the projections. These morphological characteristics are consistent with the prezoeal features observed in Stenopodidea [10, 11].

In anomuran and brachyuran decapods, the prezoeae bear plumose processes not only on the telson but also on the first and second antennae. However, unlike those observed in stenopodidean shrimps, these processes do not fully enclose the developing zoeal setae, or in some cases consist solely of a cuticular membrane without any incorporated setae. The interiors of these processes are filled with prezoeal fluid which, as suggested by scanning electron microscopy observations, is thought to function as a hydraulic skeleton, thereby maintaining process shape and mechanical

rigidity [2].

Swimming behavior

The prezoeae of stenopodidean shrimps observed in this study occasionally exhibited swimming driven by abdominal flexion, as reported for anomuran and brachyuran prezoeae. However, the predominant mode of locomotion was rhythmic movement of the second antennae. Previous research has reported that the prezoea of *St. hispidus* swims using greatly enlarged antennae bearing long setae [12]. More recent studies have likewise reported that the prezoeae of stenopodidean shrimps rely primarily on the second antennae for swimming [6, 10, 11]. The present study corroborates these earlier descriptions based on video analysis, providing direct visual evidence that antennal swimming is a characteristic and predominant behavioral trait of the prezoeal stage in Stenopodidea.

Reports of swimming behavior during the prezoeal stage in crustaceans are generally limited; however, studies on anomurans and brachyurans have shown that prezoeae do not swim using the maxillipeds, as zoeae do. Instead, locomotion is achieved through intermittent movements produced by abdominal flexion, often described as jerking behavior [17, 20, 22–24].

Prezoeae have been reported to swim by synchronous spasmodic movements of the antennules, antennae, and telson, and it has been suggested that the processes extending from these structures are involved in propulsion as well [4]. In addition, plumose setae on both the antennules and antennae of

anomuran prezoeae have been suggested to generate thrust and enable swimming activity in concert with movements of the abdomen and telson [18]. Taken together, these observations suggest that plumose processes function as oars that generate propulsive force, analogous to the natatory setae on the exopods of zoeal maxillipeds. This interpretation supports the view that the prezoea is not merely a larval form passively enclosed within a membrane, but instead possesses a relatively well-developed morphology equipped with setae and cuticular appendages. These structures appear to be specifically adapted to the characteristic swimming behavior observed during the prezoeal stage.

In the two stenopodidean species examined in this study, the prezoeae lack the well-developed cuticular processes on the antennules that are commonly observed in anomuran and brachyuran prezoeae. Instead, the antennules extend straight anteriorly from the cephalothorax and appear to be immobile. In contrast, the antennae bear a greater number of elongated processes compared with those of anomuran and brachyuran prezoeae. In addition, the antennal scale is relatively long and flexible, exhibiting considerable mobility. These morphological features indicate that stenopodidean prezoeae are structurally specialized for swimming via movements of the second antennae, which enables oriented locomotion with the cephalothorax directed toward the direction of movement.

A comparable locomotion strategy has been reported for spiny lobster (Achelata), which hatches as a phyllosoma larva. Immediately after hatching, the larvae of *Palinurus* species exhibit a morphology resembling that of a nauplius larva, a stage designated as the “naupliozoea” [25]. The

naupliozoea is characterized by a rounded body shape resulting from strong flexion and overlap of the cephalic and thoracic regions. In contrast, the abdomen is short, underdeveloped, and enclosed within a cuticular covering. At the naupliozoeal stage, the thoracic appendages which later serve as the primary swimming organs in the phyllosoma larva are folded against the body, with natatory setae remaining undeveloped, and the fourth and fifth pereopods present only as rudiments. In contrast, the second antennae are conspicuously developed and biramous, with the exopod bearing broad, plate-like swimming blades equipped with long plumose setae. It has been suggested that the naupliozoea uses vigorous movements of these highly developed second antennae to rapidly ascend to the water surface immediately after hatching. Because its mouthparts are not yet functional, the naupliozoea does not feed and undergoes molting to the subsequent phyllosoma stage within 4–6 h after hatching [25–27]. The reliance for locomotion on prominently developed antennae bearing swimming setae is a feature that naupliozoeae share with the prezoeae of stenopodidean shrimps. This similarity suggests that comparable adaptive evolutionary processes may underlie the emergence of these transient, post-hatching larval stages, as discussed below.

Duration of the prezoeal stage and its dependence on water temperature

Such temperature-dependent variation has also been documented in anomuran, astacidean, and brachyuran decapods. In some species, the embryonic cuticle is shed almost immediately upon

hatching, whereas in many others the prezoeal stage persists for several minutes up to approximately few hours before molting to the first zoeal stage (4, 17–20, 22, 28–33). Substantial variation in the duration of the prezoeal stage has also been reported in astacideans. In the abbreviated-development species *Enoplometopus occidentalis*, the prezoeal stage can last from several hours up to approximately 12 h after hatching [29]. In contrast, in the direct-developing species *Metanephrops japonicus*, the first individuals begin to molt to the megalopa stage approximately 1 h after hatching, while it might take up to 22 h for all individuals within the same clutch to complete metamorphosis [34]. These studies illustrate that the prezoeal stage can vary considerably even among individuals within a single clutch. The prezoea of the stenopodidean species *Microprosthema semilaeve* was observed to molt to the first zoeal stage within at least 6 h after hatching [13], although successful metamorphosis was observed in only 2 out of 43 individuals examined. Given this extremely low success rate, it is possible that the observed duration of the prezoeal stage reflected abnormal hatching conditions rather than a typical developmental pattern.

Temperature-dependent variation in the duration of the prezoeal stage has also been demonstrated in the naupliozoeae of achelatan lobsters. For instance, an experimental study on the effect of water temperature on *Jasus lalandii* [27] reported that 66% and 75% of naupliozoeae successfully molted to the first phyllosoma stage when they were maintained for 12 h at 5.5°C and for 15 min at 20°C, respectively. These results provide clear experimental evidence that the duration of this transient

post-hatching stage is strongly influenced by water temperature.

The mechanism through which the prezoea sheds the embryonic cuticle and transitions to the first zoeal stage remains unresolved. However, this process closely resembles molting, a phenomenon commonly observed across all crustaceans. Water temperature has been shown to significantly influence molting intervals in crustaceans [35]. For example, in Decapoda, molting is generally regulated by a decrease in the levels of molt-inhibiting hormone, followed by the release of ecdysteroid molting hormones in response to this reduction [36–38]. Elevated water temperatures may enhance metabolic activity and enzyme reaction rates associated with hormone synthesis and regulation, thus potentially accelerating the hormonal cascade controlling cuticle shedding and shortening the time required for prezoeae to shed the embryonic cuticle and complete metamorphosis into the first zoeal stage.

Biological significance of the prezoeal stage and its similarity to the pronymph stage in insects

It has been noted that, although brachyurans and some anomurans possess prominently developed spines in their the first zoeal stage, these structures are folded beneath the embryonic cuticle during the prezoeal stage [23]. This compact configuration has been suggested to facilitate passage through physical obstacles immediately after hatching, which include the dense setation of the pleopods, attached eggs, and egg stalks formed by brooding decapods to secure their egg masses. In

stenopodidean shrimps, zoeae are characterized by a long rostrum and conspicuous spines, and if these structures were fully extended immediately upon hatching, successful escape from the brood mass would likely be impeded. This observation supports the interpretation that the compact morphology of the prezoea functions as an effective escape strategy, enabling newly hatched larvae to pass smoothly through the complex physical matrix of the brood before transitioning to the more elaborate zoeal form.

As an example from symbiotic species, prezoeae of the porcellanid crab *Polyonyx gibbesi* were observed to actively escape from the host tube immediately after hatching [39]. The zoeae of porcellanid crabs possess extremely elongated spines, and hatching within the narrow confines of the host tube would therefore create a high risk of entanglement. In contrast, hatching as a prezoea with a compact body form would be an effective strategy for successful escape. A similar situation is likely to apply to spongicolid shrimps (*S. venustus* in the present study), which live symbiotically within the atrial cavity of glass sponges. Glass sponges possess mesh-like skeletal structures composed of siliceous fibers in both the body wall and the osculum, through which larvae must pass to exit the host. A zoea with fully developed spines and protrusions would probably experience substantial difficulty escaping through such structures. In contrast, the prezoea, which is characterized by the absence of fully deployed spines and a compact morphology, can be regarded as a particularly suitable form for escaping from the host environment.

Taken together, these observations suggest that the prezoeal stage may represent an important strategy during the immediate post-hatching period, facilitating both escape from physical obstacles such as egg masses or host structures and rapid ascent toward the water surface. The antenna-based swimming mode documented in this study, driven primarily by movements of the second antennae, shows a striking resemblance to the locomotor patterns observed in nauplius larvae of more basal crustaceans. This similarity suggests that the swimming behavior of prezoeae may be rooted in an ancestral propulsion pattern that has been reinforced through derived morphological specializations. In contrast to the complex and highly coordinated swimming movements involving maxillipeds during the zoeal stage, rhythmic strokes of the second antennae rely on a more limited set of movable elements. Such a locomotor mode may therefore generate a relatively simple yet powerful vertical thrust, potentially providing an efficient means of upward transport immediately after hatching.

Based on observations of hatching behavior and morphological characteristics of the prezoea in *Erimacrus isenbeckii*, it has been proposed that this stage is a phase specifically adapted for embryonic escape and is similar to the pronymph stage observed in insects [20]. The pronymph is a transient larval stage that occurs immediately after hatching in hemimetabolous insect orders such as Ephemeroptera, Odonata, Plecoptera, and Orthoptera. Morphologically, the pronymph is characterized by the entire body being enclosed within a thin and flexible cuticle, under which the

appendages remain folded and unexpanded [40–42].

The pronymph is known to shed its cuticle within a short period ranging from several minutes to a few hours after hatching, subsequently molting into the next nymphal stage [40]. The pronymph stage is generally regarded as an adaptation facilitating escape from the oviposition site or the immediate post-hatching environment [42, 43]. For example, many grasshoppers lay their eggs underground and hatch as pronymphs enclosed within a thin cuticle. These pronymphs exhibit a larva-like body form and are often referred to as vermiform larvae. Locomotion at this stage is achieved by peristaltic, worm-like movements similar to those observed in true larvae, allowing the pronymph to emerge from the soil to the ground surface. Once above ground, the pronymph sheds its cuticle and rapidly transforms into a nymph specialized for terrestrial locomotion using fully functional appendages [42–44]. In summary, by temporarily folding the appendages and adopting a larva-like body form enclosed within a thin cuticle, pronymphs can move and escape through substrates that would be difficult to traverse with the typical nymphal morphology. Locomotion relying on worm-like, peristaltic, or jerking movements allows effective displacement through complex or resistant environments.

In dragonflies, for example, pronymphs hatch from eggs deposited within plant tissues or on land and are able to move toward the water surface by vigorous abdominal flexion, repeatedly propelling the body without using the legs. Upon contact with water, they rapidly shed their cuticle and

transform into aquatic nymphs (naiad stage), a process that has been directly observed in both field and laboratory studies [43, 45].

The pronymphs in insects and the prezoeae in decapod crustaceans share three fundamental characteristics: (1) a compact body form enclosed within a cuticle (or embryonic cuticle) with folded appendages, (2) an extremely short duration, and (3) the presence of distinct behaviors specialized for movement across substrates and escape immediately after hatching. These features suggest that both stages are transient emergence phases adapted to overcoming physical barriers at the very beginning of life after hatching. From a broader evolutionary perspective, these similarities may represent an example of convergent or parallel adaptive strategies independently acquired across different arthropod lineages. Faced with comparable selective pressures—namely, the need to escape from eggs, brooding structures, host organisms, or physically complex substrates immediately after hatching—arthropods appear to have repeatedly evolved short-lived, compact, and behaviorally specialized post-hatching stages that facilitate successful emergence into the external environment.

Acknowledgments

We sincerely thank Dr. K. Tanaka and S. Yukikawa, technical staff of UMBI, as well as the members of the Laboratory of Marine Zoology, for their generous assistance in collecting ovigerous females aboard the TR/V *Toyohata-maru*. We also express our deep gratitude to Captain Tomohumi

Yamazaki and the crew of the *Tsukasa-maru*, for providing specimens, and K. Kumaki (Laboratory of Marine Biology, Kochi University) for his kind assistance. We are further grateful to the Kuroshio Biological Research Institute for their support during SCUBA-based surveys in Otsuki Town.

References

1. Williamson DI. Larval morphology and diversity. In: Bliss DE, Abele LG, editors. The biology of Crustacea. Vol 2, Embryology, morphology, and genetics. New York: Academic Press;1982. p. 43–110.
2. Quintana R, Konishi K. Ultrastructure of the prezoeal cuticle and integument of the first zoea of *Pagurus brachiomastus* (Thallwitz) (Anomura: Paguridae) just after hatching. J Nat Hist. 1988;22(4):1077–84. https://doi.org/10.1080/00222938800770661
3. Anger K. Contributions of larval biology to crustacean research: A review. Invert Repr Dev. 2006;49(3):175–205. doi: 10.1080/07924259.2006.9652207
4. Hong SY. The prezoeal stage in various decapod crustaceans. J Nat Hist. 1988;22:1041–75. doi: 10.1080/00222938800770651
5. Kalacheva NV, Ginanova TT, Kamenev YO, Maslennikov SI, Dolmatov IY. Morphology and ultrastructure of digestive system in pre-zoea and zoea I larvae

of the red king crab *Paralithodes camtschaticus*. Cell Tis Res. 2024;395:1–20. doi: 10.1007/s00441-023-03843-w PMID: 38041000

6. Martin JW, Olesen J, Høeg JT, editors. Atlas of crustacean larvae. Baltimore (MD): Johns Hopkins University Press;2014. 323 p.
7. Willemoes-Suhm R von. Briefe von R. v. Willemoes-Suhm an C. Th. E. von Siebold. Zeit Wissen Zool. 1876;26(Suppl):109–25.
8. Spence Bate C. Report on the Crustacea Macrura collected by H.M.S. Challenger during the years 1873–76. Rep Sci Res Voyage H.M.S. Challenger, Zool. 1888;24:1–942.
9. Gore RH. The larval development of the commensal crab *Polyonyx gibbesi* Haig, 1956 (Crustacea: Decapoda). Biol Bull. 1968;135:111–29. doi: 10.2307/1539619
10. Goy JW. Infraorder Stenopodidea Claus, 1872. In: Schram FR, von Vaupel Klein JC, editors. Treatise on zoology—anatomy, taxonomy, biology. The Crustacea. Vol 9, Pt A. Leiden: Brill;2010. p. 215–65.
11. Goy JW. Stenopodidea. In: Martin JW, Olesen J, Høeg JT, editors. Atlas of crustacean larvae. Baltimore (MD): Johns Hopkins University Press;2014. p. 243–9.
12. Brooks WK, Herrick FH. The embryology and metamorphosis of the Macrura.

Mem Nat Acad Sci. 1891;5:321–576.

13. Martin JW, Goy JW. The first larval stage of *Microprosthema semilaeve* (von Martens, 1872) (Crustacea: Decapoda: Stenopodidea) obtained in the laboratory. Gulf Caribbean Res. 2004;16(1):19–25. doi: 10.18785/gcr.1601.03

14. Saito T, Konishi K. Direct development in the deep-sea sponge-associated shrimp *Spongicola japonica* Kubo. J Crustacean Biol. 1999;19:46–52. doi: 10.2307/1549545

15. Gurney R. Larvae of decapod Crustacea. London: Ray Society; 1942. 306 p.

16. Saelzer HE, Quintana R, Quiñones R. Larval development of *Petrolisthes granulosus* (Guerin, 1835) (Decapoda: Anomura: Porcellanidae) under laboratory conditions. J Crustacean Biol. 1986;6(4):804–19. doi: 10.1163/193724086X00596

17. Gonor SL, Gonor JJ. Descriptions of the larvae of four North Pacific *Porcellanidae* (Crustacea: Anomura). Fish Bull. 1973;71(1):189–227.

18. Kornienko ES. Morphology of prezoea in the porcelain crab *Pachycheles stevensii* (Decapoda: Anomura: Porcellanidae) reared under laboratory conditions. Russian J Mar Biol. 2005;31(1):55–9.

19. Quintana R. On the prezoeal stage of three species of the genus *Cancer* L. from Chilean coasts (Cancridae, Brachyura). Res Crustacea 1984;13:143–51. doi:

10.18353/rcustacea.13.14.0_143

20. Sasaki J, Mihara Y. Early larval stages of the hair crab *Erimacrus isenbeckii* (Brandt) (Brachyura: Atelecyclidae), with special reference to its hatching process. J Crustacean Biol. 1993;13(3):511–28. doi: 10.2307/1548791

21. Guerao G, Abelló P. Morphology of the prezoea and first zoea of the deep-sea spider crab *Anamathia rissoana* (Brachyura: Majidae: Pisinae). Sci Mar. 1996;60(2–3):245–51.

22. Kon T. Fisheries biology of the Tanner crab, *Chionoecetes opilio*. I. On the prezoeal larva. Bull Jap Soc Sci Fish. 1967; 33(8):726–30. doi: 10.2331/suisan.33.726

23. Konishi K, Quintana R. Notes on the prezoea stage of decapod crustaceans. Aquabiology. 1987; 9: 372–8. (In Japanese with English abstract)

24. Anger K. The biology of decapod crustacean larvae. Crustacean Issues Vol 14. Lisse: A.A. Balkema;2001. 420 p.

25. Gilchrist JDF. A free-swimming nauplioid stage in *Palinurus*. J Linn Soc London, Zool. 1913;32(216):225–31.

26. Gilchrist JDF. Larval and post-larval stages of *Jasus lalandii* (Milne Edwards). J Linn Soc London, Zool. 1916;33(223):101–25.

27. Hayakawa Y, Hashitaka J, editors Minami ise ebi: Odorokubeki seitai to zōyōshoku e no chōsen = *Jasus* spiny lobsters: The wonders of their lives and the challenges to farming them. Tokyo: Seibutsu Kenkyūsha;2012. p. 286–289.

28. Okamoto K. Prezoeal stage of giant spider crab *Macrocheira kaempferi* (Crustacea, Decapoda, Majidae). Fish Sci. 1995;61(1):161–2. doi: 10.2331/fishsci.61.161

29. Okamoto K. Japanese nephropid lobster *Metanephrops japonicus* lacks zoeal stage. Fish Sci. 2008;74:98–103. doi: 0.1111/j.1444-2906.2007.01500.x

30. Davis CC. A study of the hatching process in aquatic invertebrates: XX. The blue crab, *Callinectes sapidus* Rathbun; XXI. The nemertean, *Carcinonemertes carcinophila* (Kölliker). Chesapeake Sci. 1965;6(4):201–8.

31. Hong SY, Ingle RW. Larval development of the circular crab, *Atelecyclus rotundatus* (Olivi) (Crustacea: Brachyura: Atelecyclidae) reared in the laboratory. J Nat Hist. 1987;21:1539–60. doi: 10.1080/00222938700770961

32. Motoh H. Laboratory-reared zoeae and megalopae of Zuwai crab from the Sea of Japan. Bull Jap Soc Sci Fish. 1973;39(12):1223–30.

33. Motoh H. The larval stages of Benizuwai-gani, *Chionoecetes japonicus* Rathbun, reared in the laboratory. Bull Jap Soc Sci Fish. 1976;42(5):533–42. doi:

10.2331/suisan.42.533

34. Iwata Y, Sugita H, Deguchi Y, Kamemoto FI. The larval development of reef lobster *Enoplometopus occidentalis* Randall (Decapoda, Axiidae) reared in the laboratory. Res Crust. 1991;20:1–15. doi: 10.18353/rcustacea.20.0_1

35. Zeng C, Rotllant G, Giménez L, Romano N Effects of environmental conditions on larval growth and development. In: Anger K, Harzsch S, Thiel M, editors. Developmental biology and larval ecology. Oxford: Oxford University Press;2020. p. 195–222. doi: 10.1093/oso/9780190648954.003.0007

36. Chang ES, Mykles DL. Regulation of crustacean molting: A review and our perspectives. Gen Comp Endo. 2011;172:323–30. doi: 10.1016/j.ygcen.2011.04.003 PMID: 21501612

37. Techa S, Chung JS. Ecdysteroids regulate the levels of molt-inhibiting hormone (MIH) expression in the blue crab, *Callinectes sapidus*. PLoS One 2015;10(4): e0117278. doi: 10.1371/journal.pone.0117278 PMID: 25849453

38. Mykles DL. Signaling pathways that regulate the crustacean molting gland. Front Endo. 2021;12:674711. doi: 10.3389/fendo.2021.674711 PMID: 34234741

39. Gore RH. Molting and growth in decapod larvae. In: Wenner AM, editor. Crustacean Issues 2: Larval growth. Rotterdam: A.A. Balkema;1985. p. 40–1.

40. Bernays EA. The vermiform larva of *Schistocerca gregaria* (Forskål): form and activity (Insecta, Orthoptera). Zeit Morph Tiere 1971;70:183–200.

41. Corbet PS. Dragonflies: behaviour and ecology of Odonata. Colchester (UK): Harley Books;1999. 829 p.

42. Konopová B, Zrzavý J. Ultrastructure, development, and homology of insect embryonic cuticles. J Morph. 2005;264(3):339–62. doi: 10.1002/jmor.10338

43. Truman JW, Riddiford LM. The origins of insect metamorphosis. Nature 1999;401(6752):447–52. doi: 10.1038/46737 PMID: 10442805

44. Truman JW, Riddiford LM. The evolution of insect metamorphosis: A developmental and endocrine view. Phil Trans Roy Soc B 2019;374:20190070. doi: 10.1098/rstb.2019.0070 PMID: 31438820

45. Truman JW, Riddiford LM. Endocrine insights into the evolution of metamorphosis in insects. Ann Rev Ent. 2002;47:467–500. doi: 10.1146/annurev.ento.47.091201.145230

Supporting Information

All supporting movies (S1–S4) are available for peer review via the following private Figshare link: https://figshare.com/s/7657c0198fef7ccaf70e

(This dataset will be made publicly available with a DOI upon acceptance.)

S1 Movie

Video recording made with a Nikon D5600 camera showing the transformation from prezoea to first zoea in *Spongicola venustus.*

The rostrum extends while abdominal movements pull the embryonic cuticle away from the cephalothorax and its appendages via the connecting membrane. Subsequently, while gently moving the appendages in an apparent acclimatory manner, vigorous abdominal movements enable the shedding of the embryonic cuticle covering the telson, thereby completing the transformation into the first zoea.

S2 Movie

Video recording made with a Nikon D5600 camera showing the swimming behavior of a *Stenopus hispidus* larva and its transformation from prezoea to first zoea.

The larva swims freely using the antennae. Subsequently, as observed in Movie S1 for *Spongicola venustus*, the transformation to the first zoea is completed.

S3 Movie

Video recording showing the swimming behavior of *Spongicola venustus.*

Recorded from an 8 × slow-motion movie using an iPhone 11.

S4 Movie

Video recording showing the swimming behavior of *Stenopus hispidus*.

Recorded from an 8 × slow-motion movie using an iPhone 15.

Figure 1. Hatched prezoeae of Spongicola venustus and Stenopus hispidus.

Click here to access/download;Figure;Fig.1.tif

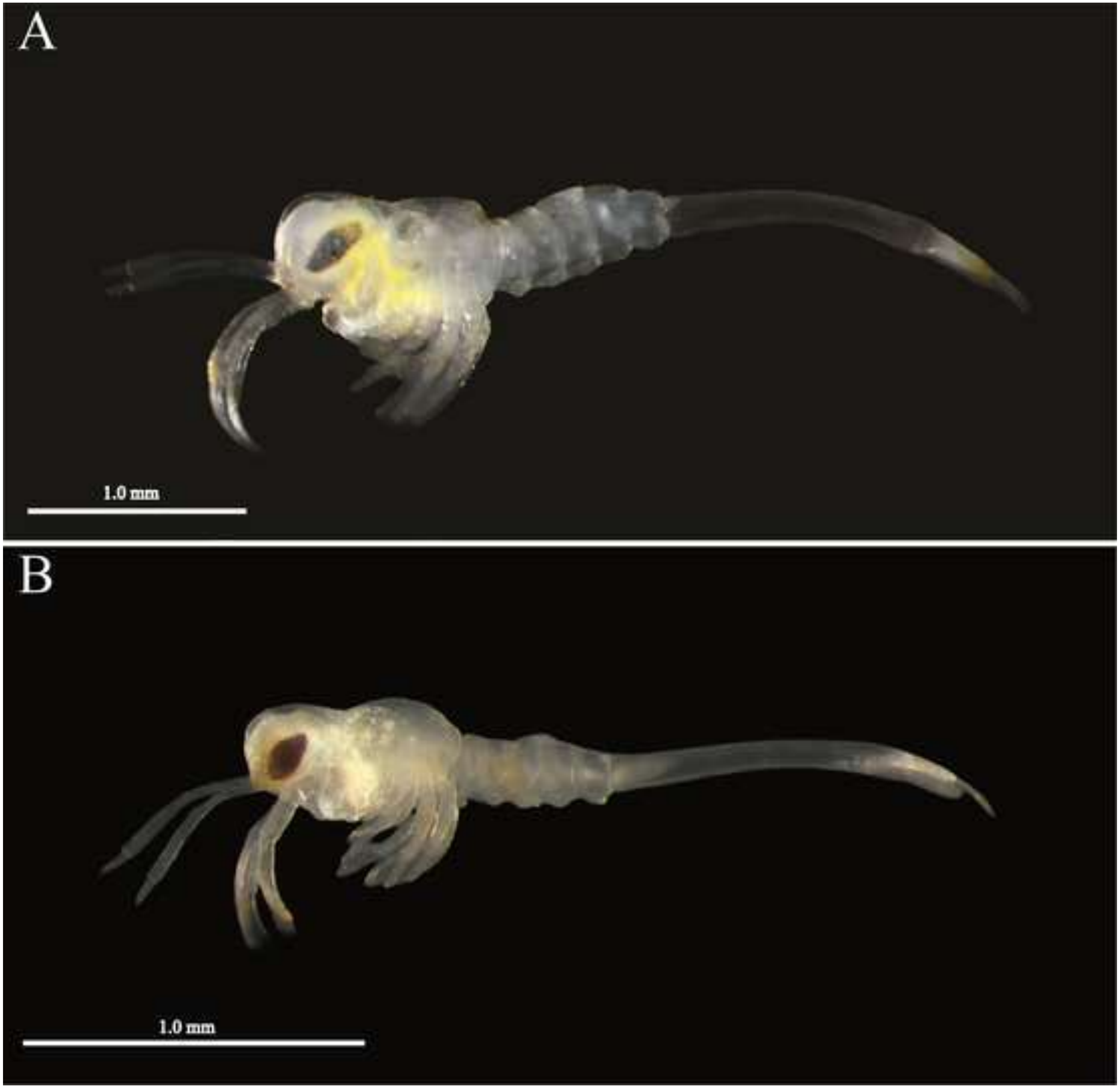

Figure 2. Duration of the prezoeal stage at different temperatures.

Click here to access/download;Figure;Fig.2.tif

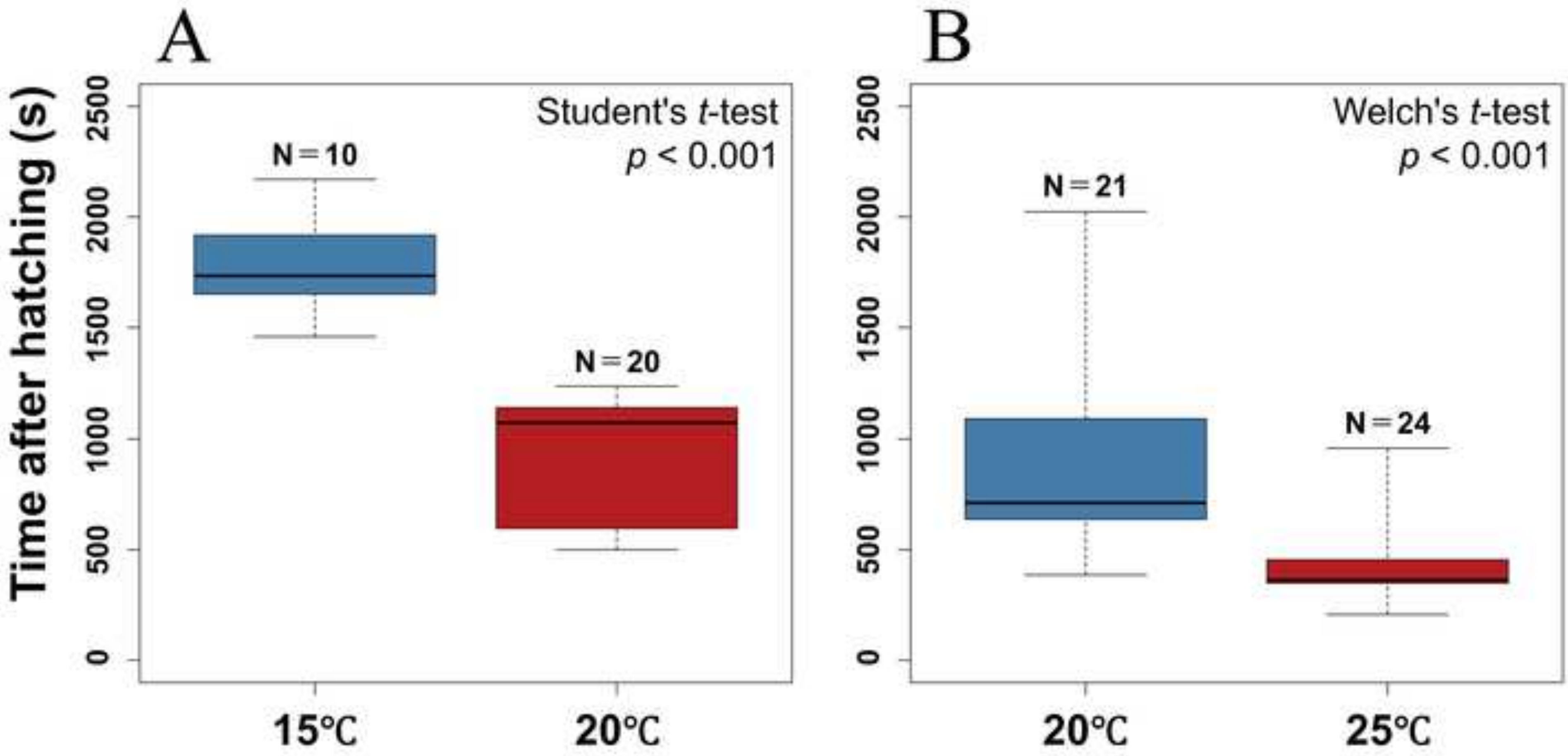

Figure 3. Morphology of prezoea in Spongicola venustus.

Click here to access/download;Figure;Fig.3.tif

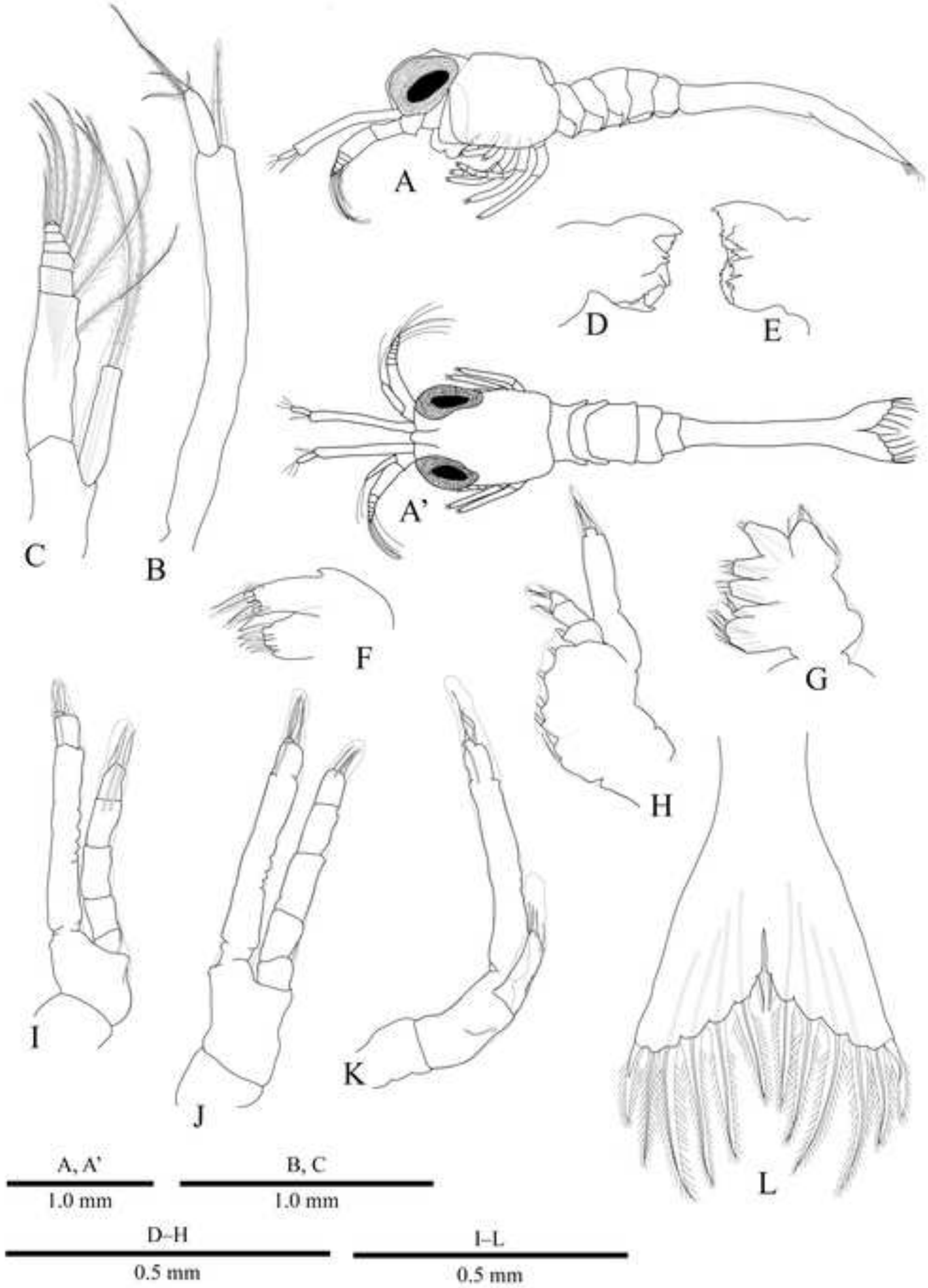

Figure 4. Morphology of prezoea in Stenopus hispidus.

Click here to access/download;Figure;Fig.4.tif

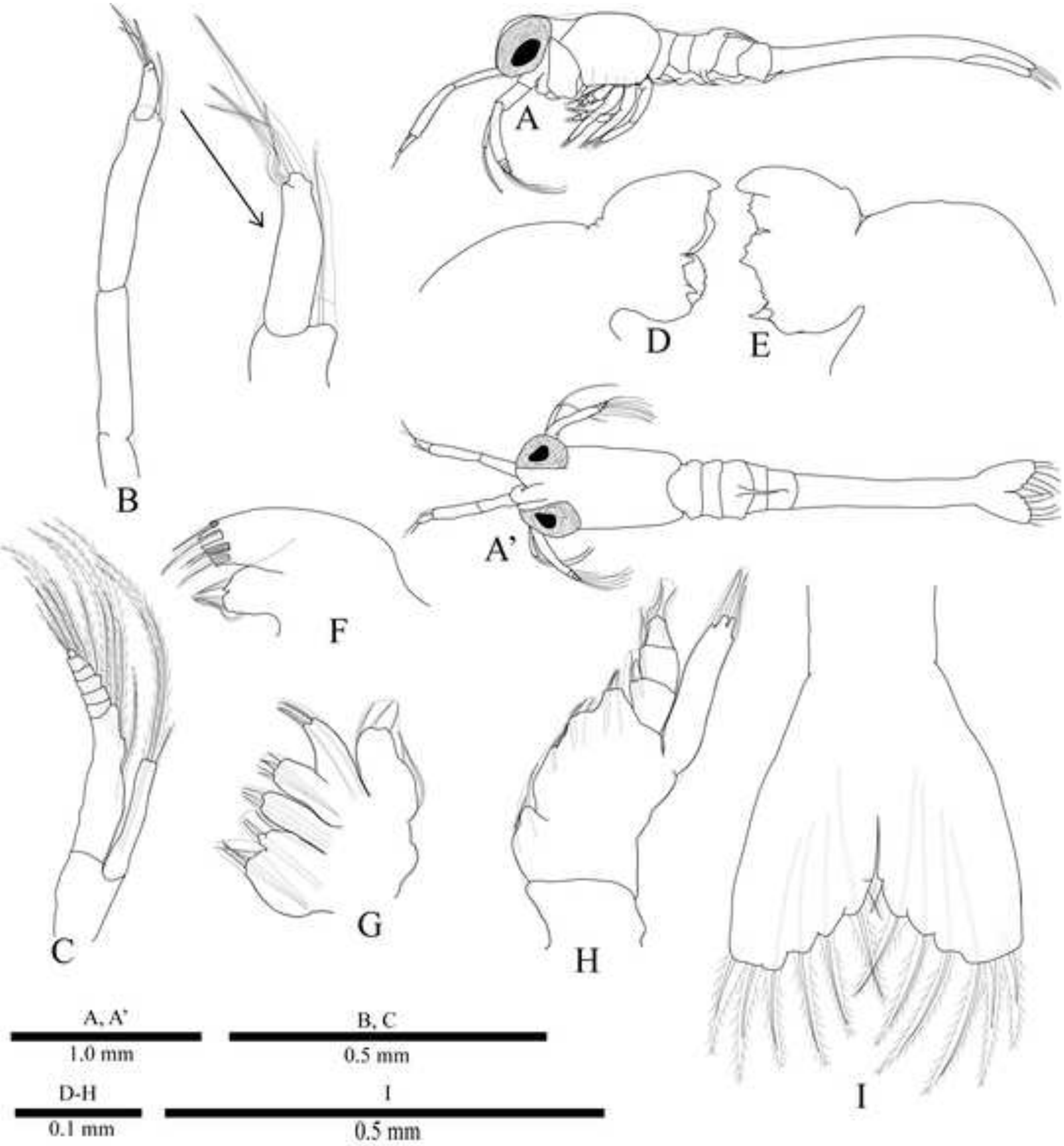

Figure 5. Appendages of prezoea in Stenopus hispidus.

Click here to access/download;Figure;Fig.5.tif

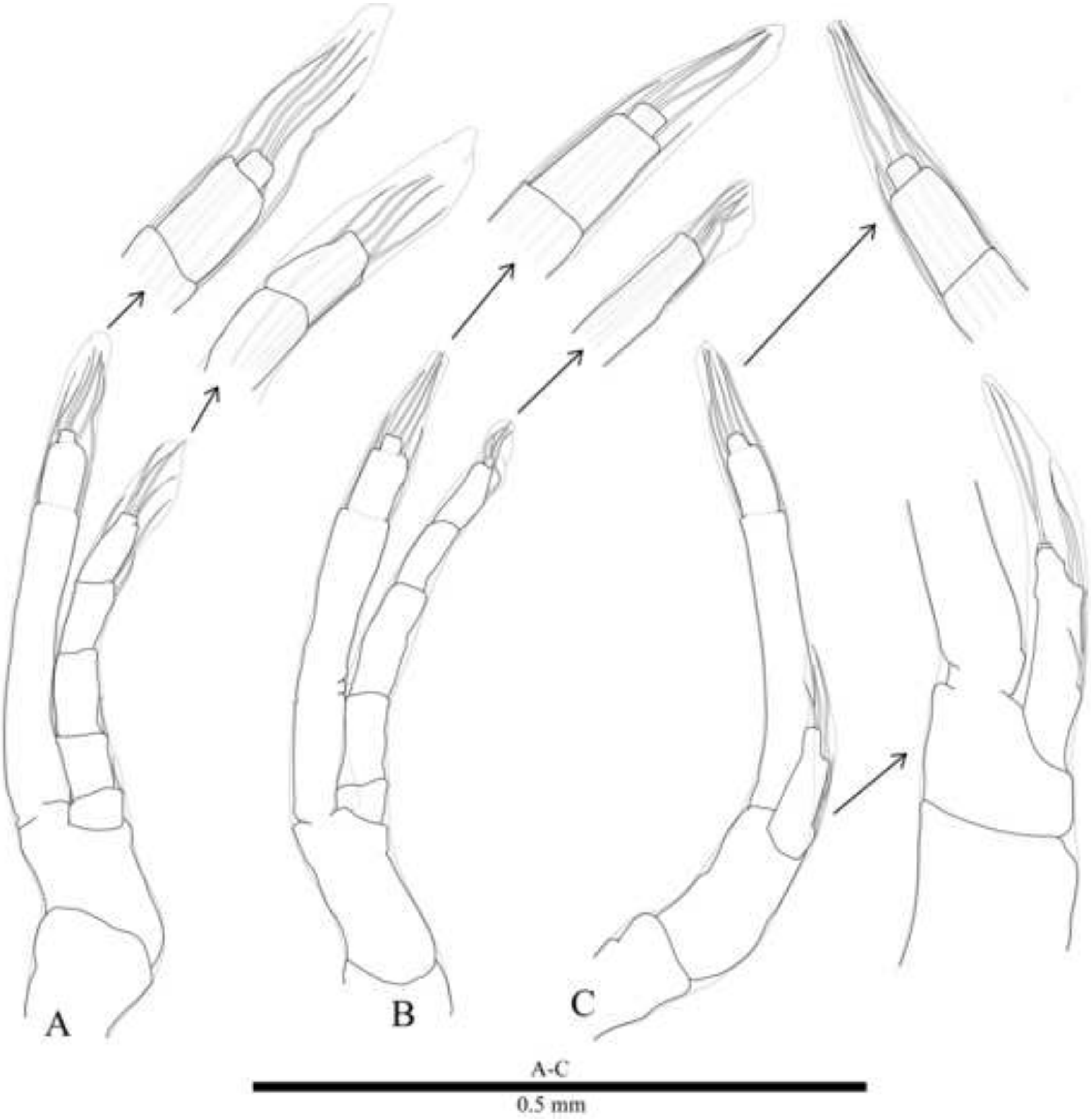

Figure 6. Swimming behavior of prezoeae.

Click here to access/download;Figure;Fig.6.tif

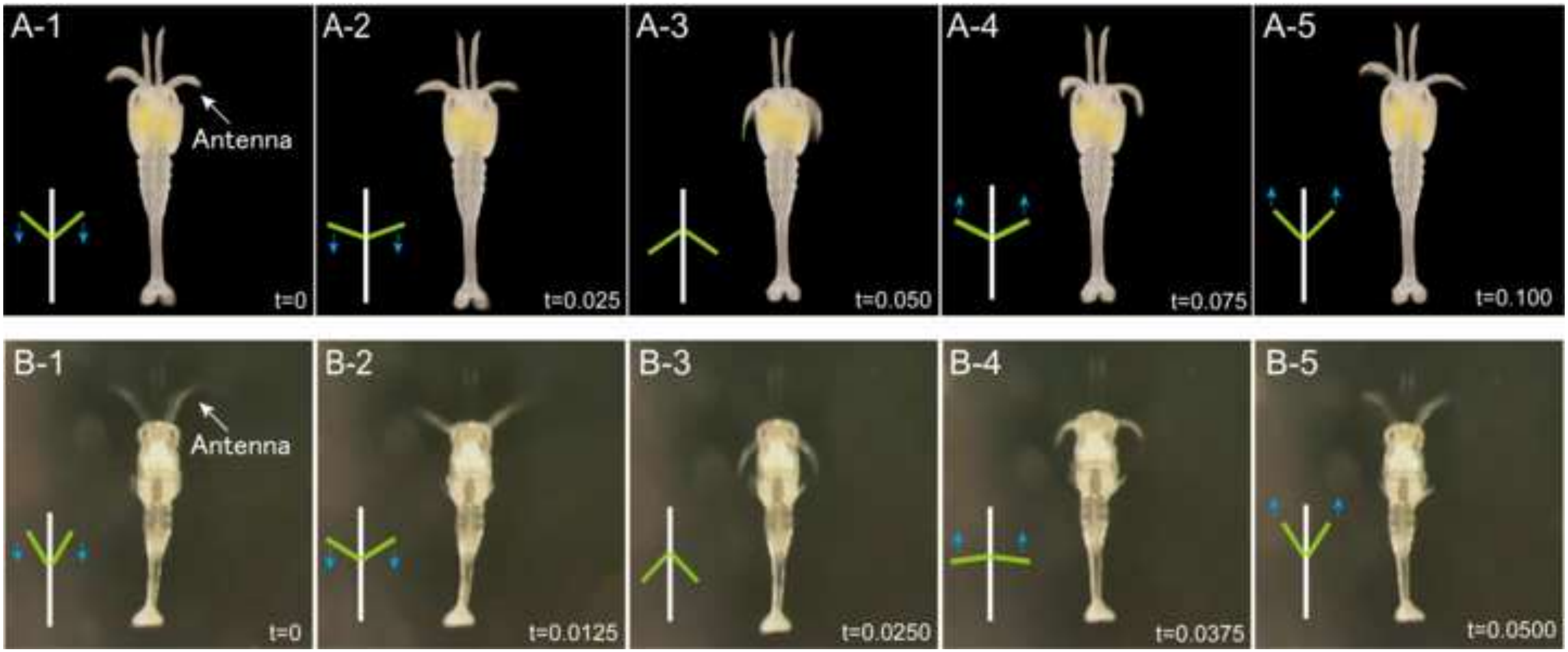

Supplement: Supplementary file 3 [file pone.0361633.s005.pdf]
