# Supplementary material for: Morphology and swimming behavior of prezoeae in two stenopodidean shrimps: Functional implications for early larval life
Source: PLoS One. 2026 Nov 10;21(11):e0361633. doi: 10.1371/journal.pone.0361633 (PMC13750586; doi:10.1371/journal.pone.0361633)
Supplement: Peer review history

# Peer review: Decision Letter 0

**DOI:** 10.1371/journal.pone.0361633.r001

**Authors:** Caio Nogueira

13 Jul 2026

Dear Dr. Nakagawa,

Thank you for submitting your manuscript to PLOS ONE. After careful consideration, we feel that it has merit but does not fully meet PLOS ONE’s publication criteria as it currently stands. Therefore, we invite you to submit a revised version of the manuscript that addresses the points raised during the review process.

Please submit your revised manuscript by Sep 11 2026 11:59PM. If you will need more time than this to complete your revisions, please reply to this message or contact the journal office at plosone@plos.org. When you're ready to submit your revision, log on to https://www.editorialmanager.com/pone/ and select the 'Submissions Needing Revision' folder to locate your manuscript file.

- A letter that responds to each point raised by the academic editor and reviewer(s). You should upload this letter as a separate file labeled 'Response to Reviewers'.
- A marked-up copy of your manuscript that highlights changes made to the original version. You should upload this as a separate file labeled 'Revised Manuscript with Track Changes'.
- An unmarked version of your revised paper without tracked changes. You should upload this as a separate file labeled 'Manuscript'.

If you would like to make changes to your financial disclosure, please include your updated statement in your cover letter. Guidelines for resubmitting your figure files are available below the reviewer comments at the end of this letter.

If applicable, we recommend that you deposit your laboratory protocols in protocols.io to enhance the reproducibility of your results. Protocols.io assigns your protocol its own identifier (DOI) so that it can be cited independently in the future. For instructions see: https://journals.plos.org/plosone/s/submission-guidelines#loc-laboratory-protocols. Additionally, PLOS ONE offers an option for publishing peer-reviewed Lab Protocol articles, which describe protocols hosted on protocols.io. Read more information on sharing protocols at https://plos.org/protocols?utm_medium=editorial-email&utm_source=authorletters&utm_campaign=protocols.

As the corresponding author, your ORCID iD is verified in the submission system and will appear in the published article. PLOS supports the use of ORCID, and we encourage all coauthors to register for an ORCID iD and use it as well. Please encourage your coauthors to verify their ORCID iD within the submission system before final acceptance, as unverified ORCID iDs will not appear in the published article. *Only*  the individual author can complete the verification step; PLOS staff *cannot*  verify ORCID iDs on behalf of authors.

We look forward to receiving your revised manuscript.

Kind regards,

Caio Santos Nogueira, PhD

Academic Editor

PLOS One

Journal Requirements:

When submitting your revision, we need you to address these additional requirements.

1. Please ensure that your manuscript meets PLOS ONE's style requirements, including those for file naming. The PLOS ONE style templates can be found at

https://journals.plos.org/plosone/s/file?id=wjVg/PLOSOne_formatting_sample_main_body.pdf and https://journals.plos.org/plosone/s/file?id=ba62/PLOSOne_formatting_sample_title_authors_affiliations.pdf

2. In the online submission form, you indicated that your data will be submitted to a repository upon acceptance. We strongly recommend all authors deposit their data before acceptance, as the process can be lengthy and hold up publication timelines. Please note that, though access restrictions are acceptable now, your entire minimal dataset will need to be made freely accessible if your manuscript is accepted for publication. This policy applies to all data except where public deposition would breach compliance with the protocol approved by your research ethics board. If you are unable to adhere to our open data policy, please kindly revise your statement to explain your reasoning and we will seek the editor's input on an exemption.

3. We note that the grant information you provided in the ‘Funding Information’ and ‘Financial Disclosure’ sections do not match.

When you resubmit, please ensure that you provide the correct grant numbers for the awards you received for your study in the ‘Funding Information’ section.

4. Please expand the acronym “JST” (as indicated in your financial disclosure) so that it states the name of your funders in full.

This information should be included in your cover letter; we will change the online submission form on your behalf.

5. We note that Figure(s) 1, 3, 4, 5, 6, in your submission contain copyrighted images. All PLOS content is published under the Creative Commons Attribution License (CC BY 4.0), which means that the manuscript, images, and Supporting Information files will be freely available online, and any third party is permitted to access, download, copy, distribute, and use these materials in any way, even commercially, with proper attribution. For more information, see our copyright guidelines: http://journals.plos.org/plosone/s/licenses-and-copyright.

We require you to either (1) present written permission from the copyright holder to publish these figures specifically under the CC BY 4.0 license, or (2) remove the figures from your submission:

a. You may seek permission from the original copyright holder of Figure(s) 1, 3, 4, 5, 6, to publish the content specifically under the CC BY 4.0 license.

We recommend that you contact the original copyright holder with the Content Permission Form (http://journals.plos.org/plosone/s/file?id=7c09/content-permission-form.pdf) and the following text:

“I request permission for the open-access journal PLOS ONE to publish XXX under the Creative Commons Attribution License (CCAL) CC BY 4.0 (http://creativecommons.org/licenses/by/4.0/). Please be aware that this license allows unrestricted use and distribution, even commercially, by third parties. Please reply and provide explicit written permission to publish XXX under a CC BY license and complete the attached form.”

Please upload the completed Content Permission Form or other proof of granted permissions as an "Other" file with your submission.

In the figure caption of the copyrighted figure, please include the following text: “Reprinted from [ref] under a CC BY license, with permission from [name of publisher], original copyright [original copyright year].”

b. If you are unable to obtain permission from the original copyright holder to publish these figures under the CC BY 4.0 license or if the copyright holder’s requirements are incompatible with the CC BY 4.0 license, please either i) remove the figure or ii) supply a replacement figure that complies with the CC BY 4.0 license. Please check copyright information on all replacement figures and update the figure caption with source information. If applicable, please specify in the figure caption text when a figure is similar but not identical to the original image and is therefore for illustrative purposes only.

6. If the reviewer comments include a recommendation to cite specific previously published works, please review and evaluate these publications to determine whether they are relevant and should be cited. There is no requirement to cite these works unless the editor has indicated otherwise.

Additional Editor Comments :

Dear Authors,

Both reviewers evaluated the manuscript and recognized its scientific merit, agreeing that it has the potential to be published. However, they also pointed out that the manuscript still requires several refinements before it is ready for publication.

Therefore, I ask that you carefully address all of the comments and suggestions provided by both reviewers when preparing your revised version.

[Note: HTML markup is below. Please do not edit.]

Reviewers' comments:

Reviewer's Responses to Questions

**Comments to the Author**

1. Is the manuscript technically sound, and do the data support the conclusions?

Reviewer #1: Yes

Reviewer #2: Yes

**********

2. Has the statistical analysis been performed appropriately and rigorously?

Reviewer #1: Yes

Reviewer #2: Yes

**********

3. Have the authors made all data underlying the findings in their manuscript fully available?

The PLOS Data policy

Reviewer #1: Yes

Reviewer #2: Yes

**********

4. Is the manuscript presented in an intelligible fashion and written in standard English?

Reviewer #1: Yes

Reviewer #2: Yes

**********

Reviewer #1: The manuscript presents a consistent and original dataset, particularly due to the combination of morphological descriptions and behavioral analyses of prezoea locomotion. However, its greatest scientific potential lies in demonstrating that this stage represents a specialized functional phase associated with emergence, initial dispersal, and survival immediately after hatching. I recommend making this concept the central focus of the Introduction and Discussion, while reducing excessive description and expanding on the biological interpretation.

The Discussion contains many descriptive paragraphs that lack actual discussion elements. I suggest a thorough revision of the Discussion, prioritizing the findings as well as the comparisons and interpretations that strengthen the argument. I have included my comments and suggestions directly in the PDF.

Special attention is required regarding the long paragraphs comparing the subject to insects. I have left some suggestions in the discussion section to avoid straying from the study's main focus. I also suggested including a hypothesis at the end of the introduction that links back to the beginning of the discussion.

Reviewer #2: The manuscript presents new and interesting data on the morphology and behavior of prezoa. The text is very well written, the methodology is clear, and the conclusions are supported by the data obtained. I only suggest that the morphological descriptions be revised, in some sections, the description does not match the figure. I have noted everything in the PDF.

**********

what does this mean?). If published, this will include your full peer review and any attached files.

If you choose “no”, your identity will remain anonymous but your review may still be made public.

**Do you want your identity to be public for this peer review?**  For information about this choice, including consent withdrawal, please see our Privacy Policy

Reviewer #1: No

Reviewer #2: No

**********

[NOTE: If reviewer comments were submitted as an attachment file, they will be attached to this email and accessible via the submission site. Please log into your account, locate the manuscript record, and check for the action link "View Attachments". If this link does not appear, there are no attachment files.]

To ensure your figures meet our technical requirements, please review our figure guidelines: https://journals.plos.org/plosone/s/figures

You may also use PLOS’s free figure tool, NAAS, to help you prepare publication quality figures: https://journals.plos.org/plosone/s/figures#loc-tools-for-figure-preparation.

NAAS will assess whether your figures meet our technical requirements by comparing each figure against our figure specifications.

Attachment Submitted filename: PONE-D-26-25140.pdf

Attachment Submitted filename: PONE-D-26-25140_reviewer with comments.pdf

# Author comment: Author response to Decision Letter 1

**DOI:** 10.1371/journal.pone.0361633.r002

7 Sep 2026

Editors' comments

If applicable, we recommend that you deposit your laboratory protocols in protocols.io to enhance the reproducibility of your results. Protocols.io assigns your protocol its own identifier (DOI) so that it can be cited independently in the future. For instructions see: https://journals.plos.org/plosone/s/submission-guidelines#loc-laboratory-protocols. Additionally, PLOS ONE offers an option for publishing peer-reviewed Lab Protocol articles, which describe protocols hosted on protocols.io. Read more information on sharing protocols at https://plos.org/protocols?utm_medium=editorial-email&utm_source=authorletters&utm_campaign=protocols.

⇒ We thank the Editor for this suggestion. We did not employ any novel or specialized experimental procedures in this study, and all methods used are described in sufficient detail in the manuscript. Therefore, we believe that deposition of a laboratory protocol in protocols.io is not necessary for the present study.

As the corresponding author, your ORCID iD is verified in the submission system and will appear in the published article. PLOS supports the use of ORCID, and we encourage all coauthors to register for an ORCID iD and use it as well. Please encourage your coauthors to verify their ORCID iD within the submission system before final acceptance, as unverified ORCID iDs will not appear in the published article. Only the individual author can complete the verification step; PLOS staff cannot verify ORCID iDs on behalf of authors

⇒ We will encourage the coauthor to verify his ORCID iD in the submission system prior to final acceptance.

１．Please ensure that your manuscript meets PLOS ONE's style requirements, including those for file naming.

⇒ We have reviewed PLOS ONE's style requirements and ensured that all manuscript formatting and file naming conventions strictly comply with the guidelines.

2. In the online submission form, you indicated that your data will be submitted to a repository upon acceptance. We strongly recommend all authors deposit their data before acceptance, as the process can be lengthy and hold up publication timelines. Please note that, though access restrictions are acceptable now, your entire minimal dataset will need to be made freely accessible if your manuscript is accepted for publication. This policy applies to all data except where public deposition would breach compliance with the protocol approved by your research ethics board. If you are unable to adhere to our open data policy, please kindly revise your statement to explain your reasoning and we will seek the editor's input on an exemption.

⇒ We have generated a DOI for the dataset on Figshare (10.6084/m9.figshare.32338614), which will become fully active and publicly accessible immediately upon publication. During the review process, the files remain accessible via the provided private link.

3. We note that the grant information you provided in the ‘Funding Information’ and ‘Financial Disclosure’ sections do not match.

When you resubmit, please ensure that you provide the correct grant numbers for the awards you received for your study in the ‘Funding Information’ section

⇒ We have updated both the Financial Disclosure statement and the Funding Information section in the online submission system to ensure consistency between them.

4. Please expand the acronym “JST” (as indicated in your financial disclosure) so that it states the name of your funders in full.

This information should be included in your cover letter; we will change the online submission form on your behalf.

⇒ We have revised the Financial Disclosure statement to provide the full name of JST as "Japan Science and Technology Agency (JST)." As requested, the revised funding statement has also been included in the cover letter.

5. We note that Figure(s) 1, 3, 4, 5, 6, in your submission contain copyrighted images. All PLOS content is published under the Creative Commons Attribution License (CC BY 4.0), which means that the manuscript, images, and Supporting Information files will be freely available online, and any third party is permitted to access, download, copy, distribute, and use these materials in any way, even commercially, with proper attribution.

⇒ We would like to clarify that all figures (Figs 1–6) are entirely original and were created solely by the authors for this manuscript.

We suspect that your concern may have arisen from the names and years shown alongside the scientific names within our figures. These represent taxonomic authority citations (e.g., Scientific name Author, Year) used according to standard zoological nomenclatural practice and do not constitute copyright notices or material reproduced from third-party publications.

Therefore, no copyrighted third-party materials are included in these figures, and all images are original works created by us and are fully compatible with publication under the CC BY 4.0 license.

6. If the reviewer comments include a recommendation to cite specific previously published works, please review and evaluate these publications to determine whether they are relevant and should be cited. There is no requirement to cite these works unless the editor has indicated otherwise.

⇒ The reviewers did not recommend any additional references for citation. Therefore, no changes were made to the reference list.

Reviewers’ comments

Reviewer 1

Major comments

１．The manuscript presents a consistent and original dataset, particularly due to the combination of morphological descriptions and behavioral analyses of prezoea locomotion. However, its greatest scientific potential lies in demonstrating that this stage represents a specialized functional phase associated with emergence, initial dispersal, and survival immediately after hatching. I recommend making this concept the central focus of the Introduction and Discussion, while reducing excessive description and expanding on the biological interpretation.

⇒ We sincerely thank for your insightful suggestion. We agree that the prezoea represents a specialized functional stage for emergence, initial dispersal, and early survival.

Accordingly, we restructured the Introduction and Discussion to focus on this conceptual framework and expanded our biological interpretations while streamlining the morphological descriptions. We believe these revisions have strengthened the focus and impact of the manuscript (rev. MS: L49–51, L74–82, L277–281, L341–346, L460–485; clean MS: L49–51, L71–80, L263–267, L302–307, L407–432).

２．The Discussion contains many descriptive paragraphs that lack actual discussion elements. I suggest a thorough revision of the Discussion, prioritizing the findings as well as the comparisons and interpretations that strengthen the argument. I have included my comments and suggestions directly in the PDF.

⇒ Following your comments, we have thoroughly revised the Discussion by removing repetitive descriptions, emphasizing key findings, and expanding comparisons with previous studies as well as their biological implication.

３．Special attention is required regarding the long paragraphs comparing the subject to insects. I have left some suggestions in the discussion section to avoid straying from the study's main focus. I also suggested including a hypothesis at the end of the introduction that links back to the beginning of the discussion.

⇒ Accordingly, we streamlined the insect-related discussion and added a clear hypothesis at the end of the Introduction that links directly to the beginning of the Discussion, thereby improving the overall logical flow of the manuscript (rev. MS: L79–82, L486–512; clean MS: L77–80, L433–459).

Reviewer 1

Minor comments

L42: The first paragraph takes a while to get to the central problem.

The first 15-20 lines are quite descriptive. The reader understands what the prezoea is, but it takes time to understand why it is important.

Right after the definition, I would add a sentence emphasizing its biological relevance:

Despite its brief duration, the prezoeal stage may represent a critical transition between embryonic and larval life, potentially influencing dispersal, locomotion, and survival immediately after hatching.

⇒ We added a sentence in the first paragraph of the Introduction (rev. MS: L49–51; clean MS: 49–51) emphasizing the potential importance of the prezoeal stage for dispersal, locomotion, and survival immediately after hatching.

L70–71: Some additional information would be:

Their contrasting habitats and life histories provide an opportunity to assess whether prezoeal morphology and behavior are evolutionarily conserved or shaped by ecological specialization.

⇒ We added the proposed sentence to clarify the rationale for comparing these two species and to emphasize the evolutionary and ecological context of the study (rev. MS: L74–78; clean MS: L71–75).

L75–78: I believe formulating a hypothesis would make the text more engaging for the reader.

perhaps a hypothesis, considering the difference in habitats

For example:

"We hypothesized that, despite their contrasting ecological niches, both species would exhibit conserved prezoeal traits, reflecting a common adaptive strategy during the transition from embryonic to larval life"

I believe the hypothesis would make an interesting connection with the first paragraph of the discussion.

⇒We added a hypothesis at the end of the Introduction stating that, despite their contrasting ecological niches, both species were expected to exhibit conserved prezoeal morphological and behavioral traits associated with emergence, early locomotion, and initial dispersal (rev. MS: L79–82; clean MS: 77–80).

L83: Spongicola venustus

L86–87: Stenopus hispidus

L94: Avoid this type of abbreviation in the text. Please revise throughout the manuscript.

⇒ All abbreviated scientific names have been fully spelled throughout the manuscript.

L194: Avoid using "The term of Abdomen"; I suggest replacing it in the text with "Pleon" or "Pleonal."

"pleonal processes"

L215: pleonal, please check in the entire text

⇒ We replaced those with “pleon” or "pleonal" throughout the manuscript.

L210–212: Prezoeae of both species swam primarily by rhythmic beating of the antennal scales (scaphocerites).

In the discussion, you would explicitly explore the idea that the use of scaphognathites as the primary propulsive structure constitutes a transitional locomotory strategy, bridging the functional gap between a newly hatched embryo—still encased in the embryonic cuticle—and the first zoea, which relies on thoracic swimming appendages. This argument directly links the behavioral results to the hypothesis that the prezoea represents a functional stage rather than merely a byproduct of hatching.

⇒ Accordingly, we expanded the Discussion to emphasize that propulsion by the enlarged antennal exopods provides a temporary locomotory mechanism until the thoracic swimming appendages become functional, thereby supporting the interpretation of the prezoea as a specialized functional stage rather than merely a byproduct of hatching (rev. MS: L370–376; clean MS: L330–336)

L216–217: This locomotor pattern differs fundamentally from that of the first zoea, which swims telson-first using natatory thoracic appendages rather than antennal propulsion.

⇒ To emphasize the fundamental difference between prezoeal and zoeal locomotion, we revised both the Results and Discussion.

In the Results, we clarified that prezoeae swim cephalothorax-first using antennal propulsion, whereas first zoeae rely on thoracic natatory appendages for sustained telson-first swimming (rev. MS: L242–247; clean MS: L229–233).

In the Discussion, we further highlighted that antennal propulsion serves as a temporary locomotory mechanism before the thoracic swimming appendages become functional, and interpreted this shift as a transitional locomotory strategy during early development (rev. MS: L370–376; clean MS: L330–336).

L231: If you choose to use a hypothesis, it must be confirmed or rejected in this first paragraph.

L234–235: the occurrence of this developmental pattern in both a deep-sea sponge-associated species and a shallow-water free-living species suggests that the prezoeal stage is evolutionarily conserved within Stenopodidea and is unlikely to represent a habitat-specific adaptation.

L262: An interpretation taking into account the different environments could give rise to an evolutionary interpretation:

The occurrence of the same swimming mechanism in both a shallow-water, free-living species and a deep-sea, sponge-associated species further suggests that antennal propulsion is an evolutionarily conserved feature of stenopodidean prezoeae.

⇒ Following the inclusion of an explicit hypothesis in the Introduction, we revised the first paragraph of the Discussion to clearly evaluate this hypothesis. We now state that the similar developmental patterns in both Spongicola venustus and Stenopus hispidus supports our hypothesis that prezoeal morphological and behavioral traits are conserved despite their contrasting ecological niches. We further emphasize that these findings suggest the prezoeal stage is evolutionarily conserved within Stenopodidea and is unlikely to represent a habitat-specific adaptation (rev. MS: L259–270, L341–346; clean MS: L245–256, L302–307).

L244–254: This paragraph does not present any discussion; while it would work well in the introduction, here—lacking comparative elements—it does not constitute a discussion. I suggest reformulating it to begin by presenting the results of the current study and contextualizing them with the information contained in the paragraph.

⇒ We reorganized this section to begin with the morphological characteristics observed in the prezoeae of Spongicola venustus and Stenopus hispidus, and subsequently compared these findings with those reported for other decapod groups. This revision places greater emphasis on the results of the present study and their broader comparative significance (rev. MS: L277–281, L287–293, L302–310; clean MS: L263–282).

L255–260: It remains a paragraph reviewing information, lacking a clear discussion or any suggestions or interpretations of the results.

⇒We revised the paragraph to include a functional interpretation of the observed cuticular sheaths, suggesting that they may protect locomotory elements during emergence while allowing their rapid deployment after metamorphosis. We think this change strengthens the discussion and links the comparative observations to the biological significance of the prezoeal stage (rev. MS: L328–331; clean MS: L289–292).

L263–271: This paragraph ends exactly where the discussion could begin. That is, it demonstrates that the results confirm previous studies but does not explore the biological significance of this locomotion, which appears to be one of the article's key findings.

L268: Because the thoracic natatory appendages remain enclosed within the embryonic cuticle at hatching, propulsion by the enlarged antennal exopods may provide an effective temporary locomotor mechanism until the first zoeal swimming apparatus becomes functional.

⇒We expanded the Discussion to interpret antennal propulsion as a functional and evolutionarily conserved locomotory strategy that facilitates early post-hatching locomotion before the thoracic swimming appendages become functional. We further discuss its role as a transitional locomotory mechanism during the shift from the prezoeal to zoeal stage (rev. MS: L341–346, L370–376; clean MS: L302–307, L330–336)

L313: Start with your results and then compare them with the literature.

Thus, it moves beyond a mere literature review to explain what its results contribute. This shift is particularly important because the effect of temperature on the duration of the prezoea stage is one of the study's few quantitative results and warrants a more in-depth discussion.

⇒ We reorganized the Discussion to begin with the quantitative results of the present study, demonstrating the strong temperature dependence of prezoeal duration in both species. We then compared our findings with previous studies on other decapod taxa and discussed their broader developmental and ecological implications. This revision places greater emphasis on the contribution of our own results and their biological significance (rev. MS: L400–410; clean MS: L357–367).

L388–389: Okay, the functional interpretation proposed here for stenopodidean prezoeae resembles the role attributed to the pronymph in several hemimetabolous insects. But I believe this part of the discussion should be reformulated by making some more specific comparisons.

L402: The discussion devotes nearly a page to the description of the pronymph before returning to its study.

L407: The comparison between prezoea and pronymph is conceptually very rich, yet it must be presented with great caution so as not to imply that you are proposing a homology or an absolute functional equivalence.

L412–414: Although pronymphs and prezoeae are not homologous developmental stages, the remarkable functional similarities suggest that comparable ecological constraints can favor the evolution of analogous transient post-hatching forms.

⇒ We substantially shortened this section and reformulated it around specific functional comparisons between pronymphs and stenopodidean prezoeae. We now explicitly emphasize that these stages are not homologous developmental stages and do not imply absolute functional equivalence. Instead, we compare shared characteristics, including a compact cuticle-covered body, folded appendages, short duration, and specialized post-hatching movements, and discuss them as analogous adaptations that may have evolved independently in response to similar ecological constraints during emergence and early survival (rev. MS: L486–512; clean MS: L433–459).

Reviewer 2

Major comments

The manuscript presents new and interesting data on the morphology and behavior of prezoa. The text is very well written, the methodology is clear, and the conclusions are supported by the data obtained. I only suggest that the morphological descriptions be revised, in some sections, the description does not match the figure. I have noted everything in the PDF.

⇒ We have thoroughly reviewed all the points noted and revised the morphological descriptions to ensure that they fully and accurately match the corresponding figures (rev. MS: L174–231; clean MS: L167–218).

Reviewer 2

Minor comments

L83: italic

⇒ The specific names have been italicized as suggested.

L160: Please, call it B and change the others.

L186: same comment of Spongicola

⇒ We have corrected the panel labels and nomenclature as suggested and ensured consistency throughout the manuscript and figures.

L160: 2-segmented peduncle? But in the figure 3b is unsegmented, please check

⇒ Re-examination of the specimen confirmed that the antennular peduncle is unsegmented, as shown in revised Figure 3C. The description in the manuscript has been corrected accordingly, and the relevant sentence has been revised to accurately reflect this morphology (rev. MS: L177–178; clean MS: L170).

L161: 2-segmented peduncle? But in the figure 3C is unsegmented, please check

L187: same comment of Spongicola

⇒ We had incorrectly described the peduncle as two-segmented in the original manuscript, whereas the figures accurately show it as unsegmented. The descriptions for both species have been corrected accordingly (rev. MS: L179, 209; clean MS: L171–172, L199–200).

L162: 7-segmented

L188–189: 7-segmented

⇒ We have corrected the descriptions for both species to indicate that the antennal exopod is 7-segmented (rev. MS: L181, L210; clean MS: L172–173, 199–200).

L169: In Figure 3H, there is no division between the coxa and the basis; in this case, it would be called a protopodite. Please check.

L195: in the Figure 4H coxa has not seta

⇒ We recognize that the original figure may have been misleading. The coxa and basis are distinguished by a slight constriction, which is indicated by the dotted line in the figure. However, an additional proximal constriction representing the boundary between the appendage and the cephalothorax may have obscured this distinction. We have therefore revised the figure to clarify these boundaries and improve interpretation (rev. Figure: 3I, 4I).

L171: coxa and basis without seta

L197: coxa and basis without seta;

⇒ We have revised the descriptions of the second and third maxillipeds in both species to indicate that the coxa and basis lack setae (rev. MS: L190–191, 219–220; clean MS: L181–182, 208)

L172: coxa and basis with 0, 1 setae, respectively

L198: coxa and basis without seta;

⇒ We have revised the descriptions of the first pereopod in both species to indicate the number of setae on the coxa and basis (rev. MS: L192, 221–222; clean MS: L182–183, 210).

L186: with peduncle

L194: 4H

L194: 5

⇒ We have corrected the peduncle description, figure citations, and numbering as suggested to ensure consistency throughout the manuscript.

L313–314: reference?

⇒ This section has been extensively revised during manuscript restructuring, and the original text has been replaced with a revised discussion based on our results and relevant literature.

Attachment Submitted filename: For_submit_Response_to_Reviewers.docx

# Peer review: Decision Letter 1

**DOI:** 10.1371/journal.pone.0361633.r003

**Authors:** Caio Nogueira

14 Oct 2026

Morphology and swimming behavior of prezoeae in two stenopodidean shrimps: functional implications for early larval life

PONE-D-26-25140R1

Dear Dr. Nakagawa,

We’re pleased to inform you that your manuscript has been judged scientifically suitable for publication and will be formally accepted for publication once it meets all outstanding technical requirements.

Within one week, you’ll receive an e-mail detailing the required amendments. When these have been addressed, you’ll receive a formal acceptance letter and your manuscript will be scheduled for publication.

An invoice will be generated when your article is formally accepted. Please note, if your institution has a publishing partnership with PLOS and your article meets the relevant criteria, all or part of your publication costs will be covered. Please make sure your user information is up-to-date by logging into Editorial Manager at Editorial Manager® and clicking the ‘Update My Information' link at the top of the page. For questions related to billing, please contact billing support.

If your institution or institutions have a press office, please notify them about your upcoming paper to help maximize its impact. If they’ll be preparing press materials, please inform our press team as soon as possible -- no later than 48 hours after receiving the formal acceptance. Your manuscript will remain under strict press embargo until 2 pm Eastern Time on the date of publication. For more information, please contact onepress@plos.org.

Kind regards,

Caio Santos Nogueira, PhD

Academic Editor

PLOS One

Additional Editor Comments (optional):

Reviewers' comments:

Reviewer's Responses to Questions

**Comments to the Author**

Reviewer #1: All comments have been addressed

Reviewer #2: All comments have been addressed

**********

2. Is the manuscript technically sound, and do the data support the conclusions?

Reviewer #1: Yes

Reviewer #2: (No Response)

**********

3. Has the statistical analysis been performed appropriately and rigorously?

Reviewer #1: Yes

Reviewer #2: (No Response)

**********

4. Have the authors made all data underlying the findings in their manuscript fully available?

The PLOS Data policy

Reviewer #1: Yes

Reviewer #2: (No Response)

**********

5. Is the manuscript presented in an intelligible fashion and written in standard English?

Reviewer #1: Yes

Reviewer #2: (No Response)

**********

Reviewer #1: All questions and comments from previous reviews have been addressed by the authors. Consequently, a significant improvement was observed in the current study. Therefore, I have no further suggestions and recommend acceptance.

Reviewer #2: (No Response)

**********

what does this mean?). If published, this will include your full peer review and any attached files.

If you choose “no”, your identity will remain anonymous but your review may still be made public.

**Do you want your identity to be public for this peer review?**  For information about this choice, including consent withdrawal, please see our Privacy Policy

Reviewer #1: No

Reviewer #2: No

**********

# Editor report: Acceptance letter

**DOI:** 10.1371/journal.pone.0361633.r004

**Authors:** Caio Nogueira

PONE-D-26-25140R1

PLOS One

Dear Dr. Nakagawa,

I'm pleased to inform you that your manuscript has been deemed suitable for publication in PLOS One. Congratulations! Your manuscript is now being handed over to our production team.

At this stage, our production department will prepare your paper for publication. This includes ensuring the following:

* All references, tables, and figures are properly cited

* All relevant supporting information is included in the manuscript submission,

* There are no issues that prevent the paper from being properly typeset

You will receive further instructions from the production team, including instructions on how to review your proof when it is ready. Please keep in mind that we are working through a large volume of accepted articles, so please give us a few days to review your paper and let you know the next and final steps.

Lastly, if your institution or institutions have a press office, please let them know about your upcoming paper now to help maximize its impact. If they'll be preparing press materials, please inform our press team within the next 48 hours. Your manuscript will remain under strict press embargo until 2 pm Eastern Time on the date of publication. For more information, please contact onepress@plos.org.

You will receive an invoice from PLOS for your publication fee after your manuscript has reached the completed accept phase. If you receive an email requesting payment before acceptance or for any other service, this may be a phishing scheme. Learn how to identify phishing emails and protect your accounts at https://explore.plos.org/phishing.

If we can help with anything else, please email us at customercare@plos.org.

Thank you for submitting your work to PLOS One and supporting open access.

Kind regards,

PLOS One Editorial Office Staff

on behalf of

Dr. Caio Santos Nogueira

Academic Editor

PLOS One
